# Age-related decrease in motor contribution to multisensory reaction times in primary school children

**DOI:** 10.3389/fnhum.2022.967081

**Published:** 2022-09-08

**Authors:** Areej A. Alhamdan, Melanie J. Murphy, Sheila G. Crewther

**Affiliations:** ^1^Department of Psychology and Counselling, La Trobe University, Melbourne, VIC, Australia; ^2^Department of Psychology, Imam Mohammad Ibn Saud Islamic University, Riyadh, Saudi Arabia; ^3^Centre for Human Psychopharmacology, Swinburne University of Technology, Melbourne, VIC, Australia

**Keywords:** multisensory integration, children, motor reaction times, auditory, visual, audiovisual, visuomotor, inspection time

## Abstract

Traditional measurement of multisensory facilitation in tasks such as speeded motor reaction tasks (MRT) consistently show age-related improvement during early childhood. However, the extent to which motor function increases with age and hence contribute to multisensory motor reaction times in young children has seldom been examined. Thus, we aimed to investigate the contribution of motor development to measures of multisensory (auditory, visual, and audiovisual) and visuomotor processing tasks in three young school age groups of children (*n* = 69) aged (5−6, *n* = 21; 7−8, *n* = 25.; 9−10 *n* = 18 years). We also aimed to determine whether age-related sensory threshold times for purely visual inspection time (IT) tasks improved significantly with age. Bayesian results showed decisive evidence for age-group differences in multisensory MRT and visuo-motor processing tasks, though the evidence showed that threshold time for visual identification IT performance was only slower in the youngest age group children (5−6) compared to older groups. Bayesian correlations between performance on the multisensory MRT and visuo-motor processing tasks indicated moderate to decisive evidence in favor of the alternative hypothesis (BF_10_ = 4.71 to 91.346), though not with the threshold IT (BF_10_ < 1.35). This suggests that visual sensory system development in children older than 6 years makes a less significant contribution to the measure of multisensory facilitation, compared to motor development. In addition to this main finding, multisensory facilitation of MRT within race-model predictions was only found in the oldest group of children (9−10), supporting previous suggestions that multisensory integration is likely to continue into late childhood/early adolescence at least.

## Introduction

Multisensory processing, when defined as the neural interaction of multiple streams of information resulting in faster processing speed, is closely associated with higher-level perceptual and cognitive tasks (Rose et al., 2008; Stein et al., 2010; Barutchu et al., 2018, 2020; Denervaud et al., 2020; Pecher and Zeelenberg, 2022). It is assumed to be responsible for improving perception, such as an enhanced accuracy of sensory estimates, and facilitating motor actions, leading to quicker and more accurate motor responses (Barutchu et al., 2009; Downing et al., 2015; Nardini et al., 2016). Traditionally, experimental testing of multisensory motor performance has utilized speeded motor reaction tasks (MRT) (e.g., Barutchu et al., 2009; Nardini et al., 2016; Ainsworth et al., 2021; Crosse et al., 2022) to demonstrate the effect of multisensory integration on motor actions in children. However, despite the well accepted age related improvement in MRT during childhood, the extent to which motor function development *per se* influences age-related performance on multisensory tasks in children has not been thoroughly investigated. Thus this study aimed to examine the contribution of age related motor performance to multisensory motor reaction times in young early school age children.

Todd (1912) was one of the earliest of many manual and saccadic reaction time investigations of the facilitatory effect of multisensory integration in adults (e.g., Raab, 1962; Miller, 1982) showing that multisensory stimuli induced faster motor responses than either unisensory stimulus alone. Raab originally suggested that the race between the senses led to the emergence of multisensory facilitation, wherein the faster sense always initiates the motor responses (Raab, 1962). However, the fact that motor responses to multisensory audiovisual stimuli were faster than responses to either audio or visual stimuli alone led to the proposal of race model inequality by Miller (1982). The race model states CDF*_*AV*_* ≤ CDF*_*A*_* (t) + CDF*_*V*_* (t), where “CDF” is the Cumulative Density Function of the audiovisual motor responses condition (AV) and individual unisensory auditory (A) and visual (V) stimuli (for more details, see Ulrich et al., 2007). Studies employing this “inequality model” have now been widely utilized in adults and indicate that the level of gains from multisensory stimuli is too high to be predicted by race models *in adults* (e.g., Giray and Ulrich, 1993; Forster et al., 2002; Couth et al., 2018; Pinto et al., 2021). By comparison, Barutchu et al. (2009) confirmed that the motor responses of children under 8 fit with the predictions of race model inequality. Thus, we aimed to evaluate and compare the development in psychophysical performance of three different age groups of young school children, on a simple multisensory task similar to that, while also utilizing the race model predictions regarding aspects of unisensory (auditory and visual) and multisensory (audiovisual) stimulus processing and degrees of MRT facilitation. This was done to ascertain whether age-related motor performance or age-related threshold times for purely visual inspection (IT) tasks that measure object recognition time without any motor reaction aspects, also improve significantly with age.

Recent research has also shown that race models together with time for switching attention between sensorily driven information channels [i.e., the Modality Shift Effect (MSE) (Poole et al., 2021)] systematically contribute to multisensory motor speed enhancements (Fairhall and Macaluso, 2009; Botta et al., 2011; Barutchu et al., 2019; Barutchu and Spence, 2020). MSE also suggests that when individuals change tasks between uni-, and multisensory trials that require concurrent shifts in attention across or within the senses (Alsius et al., 2005; Kiesel et al., 2010; Talsma et al., 2010) then such a shift in attention and consciousness is predominantly reflected in slower and less accurate performance than automatic repetition of the same task (e.g., Sandhu and Dyson, 2013; Liu and Otto, 2020). Indeed, switching in stimulus-response domains may also confound changing the sensory modality and thus affect RT responses and motor speed performance (Otto and Mamassian, 2012; Shaw et al., 2020) especially in multisensory processing of unfamiliar stimuli in children.

Most multisensory processes, that are initiated by simple bottom-up information rapidly become familiar and automatized with practice, are underpinned and constantly being modulated by multiple neural networks including many subcortical brain regions as well as primary cortical and multiple secondary sensory processing areas (Pickering et al., 2021). Neurons responsive to information from multiple sensory systems have been identified in the brainstem Superior Colliculus (SC) region (Stein and Meredith, 1993; Driver and Noesselt, 2008) and in the thalamic pulvinar nucleus (Andersen et al., 1997; Driver and Noesselt, 2008; Froesel et al., 2021). More specifically, vision and audition have direct projections to the human colliculi and thalamic regions that are linked to the motor and frontal cortex (Corbetta and Shulman, 2002; Knopfel et al., 2019). Indeed, a behavioral and event-related potentials (ERPs) developmental study conducted by Brandwein et al. (2011) found that there was an overall positive correlation between age-group and brain processes underlying multisensory integration using motor speed audiovisual tasks. The behavioral data of this study also revealed that multisensory facilitation of simple motor reaction time tasks continues to develop till late adolescence.

Together, these lines of evidence provide direct support for the significant contribution of motor development to measurement of multisensory facilitation, while highlighting the different rates at which the motor responses and processing of different sensory information appear to develop. Thus, we aimed to investigate whether motor development is a limiting contributor to the current consideration of multisensory facilitation by assessing different tasks of motor functions associated with audiovisual and visuo-motor processing in elementary school children aged 5−10 years. Our rationale was based on the studies of Barutchu et al. (2009); Brandwein et al. (2011) who both found longer MRTs on the unisensory visual task compared to the unisensory auditory tasks even among adults. Consequently, we sought to investigate whether the improvement in multisensory facilitation is more closely related to motor development or vision alone. To do this, we used a simple motor detection task (audiovisual detection paradigm), similar to previous studies with children and adults by Barutchu et al. (2009) compared to a motor free assessment of threshold time for visual detection and identification of a stimulus in progressively shortened presentation times. An eye hand co-ordination task was also used to measure time for a child using their preferred hand to perform a more cognitive visually driven goal-directed tracing of a presented shape using the “SLURP” iPad app (Lee et al., 2014). This task is recognized as requiring integrated concurrent neural processing of vision, cognition, and manual dexterity (Wijesundera et al., 2022).

Lastly to determine the role visual development alone, played in threshold time for object/shape identification without a motor component we utilized the visual Inspection Time (IT) task, where improvements in IT performance has previously been used to predict future abilities on cognitive tasks such as perceptual speed, verbal IQ and working memory (Nettelbeck and Young, 1990; Nettelbeck and Wilson, 2004; Brown and Crewther, 2017; Ebaid et al., 2017). A modified Inspection Time (IT) task, was based on the version of Vickers et al. (1972) and adapted by Brown and Crewther (2017) for children aged 7–11 years.

To date little is known about the motor development in multi and unisensory tasks (i.e., simple detection task, and visuomotor processing) compared to non-motor visual perceptual time and how these abilities may develop with age. Therefore, the primary aims of the present study were:

(i) to examine age-related differences in motor reaction times to unisensory (visual and auditory), multisensory (audiovisual), and visuo-motor processing compared to non-motor visual perceptual processing.
(ii) to determine the relationships between the facilitation of classically defined multisensory integration, visuomotor, and non-motor visual perceptual processing.

We hypothesized that the children categorized as age groups of 5−6 years, 7−8 years, and 9−10 years old, would demonstrate significant age related improvements in motor reaction times as the older group would outperform younger groups on the processing of unisensory (auditory and visual) and multisensory information (audiovisual) and for the visuomotor task. We expected that facilitation of MRT associated with detection of simple unisensory and multisensory stimuli would correlate more significantly with all motor measures (Barutchu et al., 2009; Nardini et al., 2016) than threshold times for a simple unisensory vision system task.

## Materials and methods

### Participants

The present study included three groups of children: 5−6 years (*n* = 25), 7−8 years (*n* = 26), and 9−10 years (*n* = 18) recruited from Catholic and Public Elementary Schools in Victoria, Australia where all children from Prep/Foundation to Grade 4 were invited to participate in this project. Conduct of the project was approved by the Victorian Department of Education and the individual School Principals who facilitated in distribution of information and ethical consent forms to all student parent/guardians. This study was approved by the La Trobe University Human Ethics Committee (HEC 18139) and the Victorian Department of Education Human Ethics Committee, and the Victorian Catholic Schools Ethics Committee.

**FIGURE 1:**
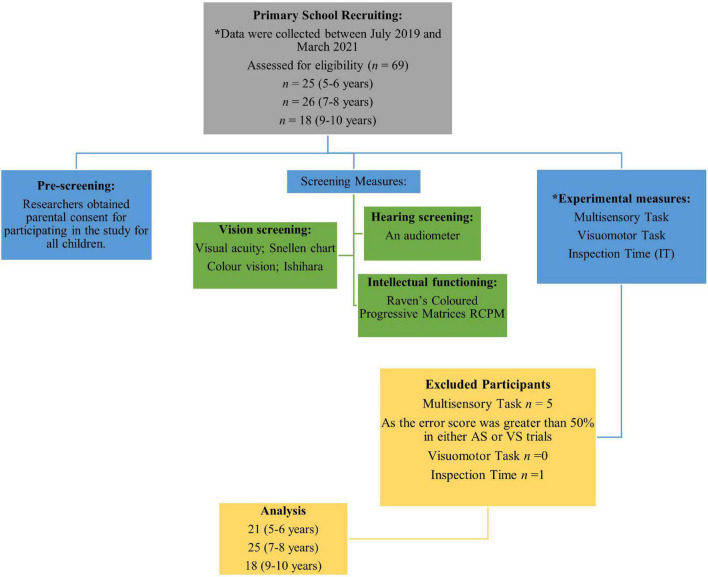
Flowchart illustrating participant groups, eligibility criteria and experimental series. *There was no set task order after screening measures.

Only children whose parents had returned signed forms indicating parental agreement to “my child may participate in the project,” and parental completion of the accompanying brief questionnaire relating to medical health and neurodevelopmental anomalies were included in the study. Only children aged 5−10 years, with normal or corrected to normal vision and hearing, no history of a neurodevelopmental disorder (e.g., ADHD, ASD, Language Disorder or Intellectual Disability and scores below the 16th percentile, i.e., more than one standard deviation below the mean) on the Raven’s Coloured Progressive Matrices test of non-verbal intelligence were excluded in analyses for this study. Children and parents were informed that they could withdraw their child from the study at any time, in accordance with the Declaration of Helsinki. Prior to testing sessions, verbal assent was also ascertained from each child. The participant groups, eligibility criteria and experimental series flowchart are shown in Figure 1.

### Screening measures

#### Vision and hearing screening

All children were screened for normal hearing, and normal or corrected-to-normal visual acuity. Vision screening included the assessment of distance and near and visual acuity using a Snellen chart, and the Ishihara test of color vision. Hearing threshold was tested using a commercial audiometer (Interacoustic Screening Audiometer, portable audiometer model AS208) to evaluate hearing acuity, with a Peltor H7A sound-attenuating headphones to assess the ability to hear sounds with frequencies ranging from 250 Hz to 8000 Hz and the sound pressure level SPL at 20 dB at each octave step. Hearing Screening was measured for both ears with the child being required to indicate each time they hear the sound (the tone’s intensity was reduced) by raising the hand on same side as sound and placing it down when stops. The hearing screening procedure was carried out in accordance with the Guideline for Hearing Screening in the School Setting, Missouri Department of Health and Senior Services Division of Community and Public Health.

#### Intellectual functioning

Non-verbal IQ was measured with Raven’s Coloured Progressive Matrices RCPM (Raven and Court, 1990) for all participants. This test was selected as performance requires visual cognitive manipulation rather than prescribed auditory/lexicon choices. The RCPM is a quick and well normed psychometric test for non-verbal reasoning abilities normed in the United Kingdom (Raven et al., 1988) and in Australian children aged 5−11 years (Cotton et al., 2005) and is accepted internationally as a valid and reliable representation of non-verbal intellectual ability. The untimed test consists of 36 items divided into three sets (A, Ab, B) each comprising 12 problems. These sets listed in order of difficulty and complexity within each subset. Four distinct intellectual abilities are measured by the RCPM: (1) Completion of Simple Continuous Patterns, (2) Completion of Discrete Patterns, (3) Continuity and Reconstruction of Simple and Complex Structures, and (4) Reasoning by Analogy (Corman and Budoff, 1974; Goharpey et al., 2013). There are six possible alternatives available for each test item, and the participant is asked to identify the appropriate option to complete the matrix pattern.

### Experimental measures

#### Multisensory task

**FIGURE 2:**
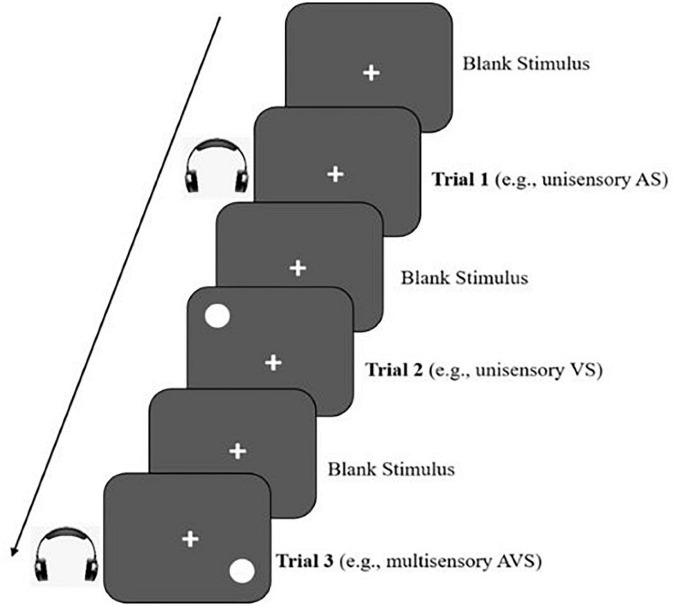
An example of a sequence of multisensory task trial showing each of the stimuli type.

Multisensory processing thresholds were measured as motor reaction times to target detection similar to the procedure used by Barutchu et al. (2009). The stimuli were presented and controlled using VPixx™ software (V 3.20), and RESPONSEPixx (VPixx, Vision Science Solutions, Quebec, Canada). The children were presented with either: an auditory stimulus (AS; *beep)*, a visual stimulus (VS; *gray circle)*, an audiovisual stimulus (AVS; *beep and gray circle presented simultaneously)*, or a blank invalid trial (see Figure 2). The AS had a tone of 1500-Hz presented through closed headphones with a rise and fall time of 5ms. The visual stimuli were presented non-centrally with the target location varying between trials, and hence requiring greater attentional demands for successful completion. Visual stimuli were never positioned centrally and always presented peripherally as a Gaussian circle enveloped with sinusoidal shading across the stimuli. The children were instructed to use the forefinger of preferred hand to press on the RESPONSEPixx handheld 5-button response box that was made by Vpixx and developed by Peter April^1^. In our task, the (down-blue-button) has been chosen to indicate whether and when a stimulus had appeared, and to record their response for each trial as rapidly and accurately as possible. Practice trials for each condition (AS, VS, AVS, and blank trial) were presented till participants were familiar with the task and performing accurately and rapidly to ensure that even the youngest children understood the procedures prior to testing. Each child completed a total of 60 test trials in 4 blocks of 15 trials of each stimulus presented in random order. Two variables were extracted from each of the multisensory task condition (AS, VS, and AVS); time taken [i.e., mean motor reaction times (MRTs) between the onset of the stimulus and the pressing of the response button, and accuracy, i.e., number of errors made by each participant for each stimulus]. All trials were presented following an interstimulus interval (ISI) between 1500 and 2500 ms with a duration of 150 ms. RTs scores within 150–1500 ms were used to calculate the mean RT for participants. Error rates lower than 50% (i.e., seven out of 15 errors) for either AS or VS were excluded. As a measure of internal reliability the Cronbach alpha of multisensory task, was calculated for a total score of 0.93 and ranged between 0.87 to 0.9 for the AS, VA and AVS, indicating high reliability (Gliem and Gliem, 2003).

#### Visuomotor processing using the SLURP (eye-hand coordination app)

**FIGURE 3:**
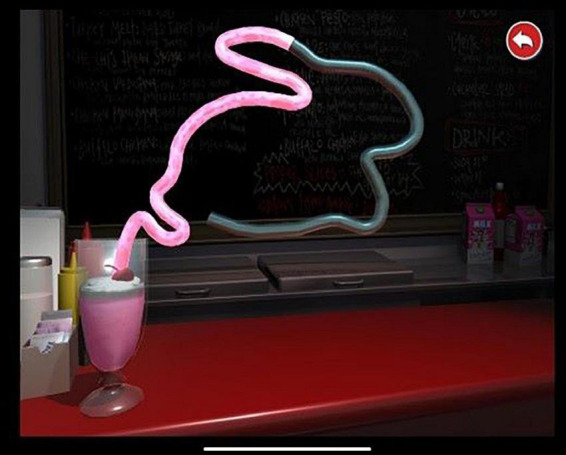
Lee Ryan Hand Co-ordination Test (SLURP).

The Lee-Ryan Eye-Hand Coordination Test Battery (SLURP) (Lee et al., 2014) iPad^®^ application is a measure of fine visually driven motor (visuo-motor) processing in both children Alghamdi et al. (2021) and adults (Junghans and Khuu, 2019). This task has demonstrated the reliability and validity to assess visuomotor integration (Lee et al., 2014; Junghans and Khuu, 2019). In this task, children were asked to trace five shapes in order (*Circle, Triangle, Square, Rabbit, and Snail*) with their fingers as fast and accurately as possible (see Figure 3). The time taken and the number of errors made to complete each shape were automatically recorded. Total time scores are an important consideration when undertaking any eye-hand coordination (Miall and Reckess, 2002; Jung and Haier, 2007), thus only total time scores were extracted and analyzed from this task. Children first completed the practice trial with a Castle shape in order to ensure the familiarity with all aspects of the task (Lee et al., 2014). This item was chosen as it is comparatively difficult to trace and requires several angled and round changes of direction across a 12-inch iPad screen long distance.

#### Inspection time visual perceptual processing task

**FIGURE 4:**
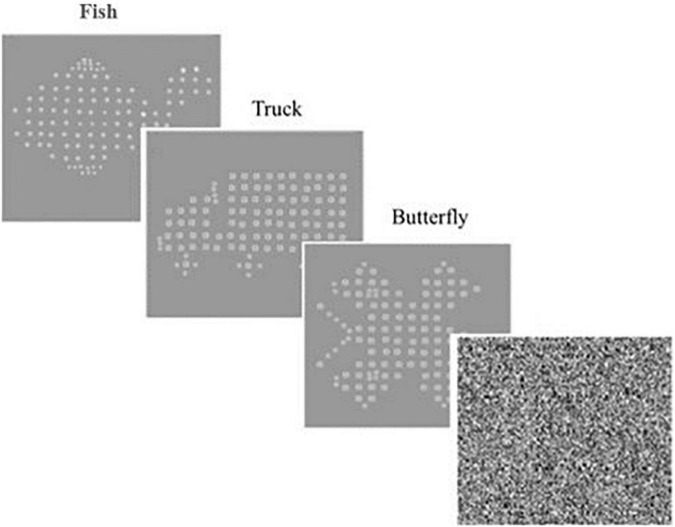
Inspection time (IT) trials *Fish, Truck and Butterfly*, only one stimulus presented for each trial.

An Inspection Time (IT) task was used as a non-motor assessment of the speed of visual processing, based on a modified Vpixx version of Vickers et al. (1972) by Brown and Crewther (2017). In this task, three simple stimuli either (a *Fish*, *Truck* or *Butterfly)*, were presented on an iMac computer (see Figure 4). A total of 32 trials and three practice trials (or more, where needed) were performed for each child. Estimation of exposure duration and confidence intervals (CI) were calculated using a parameter estimation by sequential testing (PEST) procedure, where the shorter exposure durations indicate a faster rate of visual information processing. Confidence intervals of 80% or below were extracted (Ebaid et al., 2017). Shorter exposure durations indicate a faster rate of visual information processing. IT task has been shown to be valid and reliable measure of visual information processing (Brown and Crewther, 2017; Ebaid et al., 2017).

### Procedure

All children were tested individually in a quiet unused classroom during school hours across four sessions, with at least two researchers present. Sessions were restricted to 20 and 30 mins to ensure that children’s performances were not affected by fatigue or loss of motivation. Children were free to take breaks or leave at any time. Participant’s vision and hearing were assessed at the beginning of the first session. Parents were given feedback on all hearing and sight measures and where appropriate referrals were made. Where parents agreed to schools being informed of child’s performance this information was also made available. Participants were presented with practice trials prior to the beginning of each experimental task. At the end of each session, the children were thanked for their participation and received their choice of a small stationary item.

### Data analysis

#### Power analysis

An *a priori* power analysis conducted using the G*Power 3.1 (Faul et al., 2007) indicated that a sample of 36 participants was required to obtain a moderate effect size at α < 0.05 at power of 0.8 (1-β error probability) for one way ANOVAs. However, the specific power outcome relating to each ANOVA showed that we have achieved and exceeded this power as we obtained a power of ≥0.9 (1-β error probability) (Cohen, 1992).

#### Data cleaning and outliers

The MRT response for each participant was recorded and averaged. As per previous publications (Barutchu et al., 2009; Ostrolenk et al., 2019), responses with reaction time values below 150 ms or greater than 1500 ms were excluded. It was assumed that extremely slow RTs were an indication of participant inattention, while overly fast RTs usually indicated either a response to a previous stimulus or a false alarm. Overall, only 1% of the RTs responses based on this criteria were excluded. Blank stimuli of multisensory task were not included in data analysis. The data of 5 children (four in the 5−6 years group, one in the 7−8 years group) whose error score was greater than 50% in either the AS or VS trials were excluded. For the IT task, one outlier was identified using boxplots and was removed from analysis; none were found for the SLURP task. For frequentist statistics presented in Supplementary materials, the assumptions of normality, linearity and homogeneity of variances were checked and not violated. The participants were divided into three categorical age groups (5−6, 7−8, and 9−10 years) bearing in mind that each age group represents the median school class age in Victorian schools. NVIQ was measured for all participants to ensure that children were within normal IQ range. Age and NVIQ data for each group are presented in Table 1.

**TABLE 1:** Descriptive statistics of the mean age (±SD) and IQ raw scores measure for each age group.

		Age			Non-verbal IQ		
*N*		Min.	Max.	M ± SD	Min.	Max.	M ± SD
5−6 years	25	5.06	6.90	6.00 ± 0.57	11.00	29.00	18.52 ± 0.67
7−8 years	26	7.00	8.79	7.92 ± 0.48	20.00	34.00	26.69 ± 0.73
9−10 years	18	9.00	10.97	9.86 ± 0.68	27.00	34.00	29.84 ± 0.71
Total	69						

Age is presented in “years, months.”

#### ANOVA

A series of Bayesian one-way ANOVAs (BF_10_) were conducted to determine whether performance on the MRT measures, i.e., multisensory task “AS, VS and AVS,” visuo-motor “SLURP,” and non-motor visual perpetual processing “IT” were significantly different between the three age groups. These analyses were followed by pairwise *post hoc* Bayesian *t*-tests with default Cauchy prior as appropriate. A higher Bayes factors (BF_10_) ratio indicates evidence in favor of the alternative hypothesis (H_1_) relative to the null (H_0_) (Nuzzo, 2017). For interpretation of BF_10_ the following classifications were used; 1−3 indicates anecdotal evidence, 3−10 moderate evidence, 10−30 strong evidence, 30−100 very strong evidence, and >100 as decisive evidence (Wetzels and Wagenmakers, 2012). The posterior odds, and 95% credible intervals (95% CI) were reported. We calculated also Omega-squared (ω^2^) for ANOVA to estimate the effect size ES for the differences between our groups to ensure less biased estimations of variance across aspects of the design (Olejnik and Algina, 2003; Lakens, 2013). Effect sizes were set at: ω^2^ > 0.01 = small; ω^2^ > 0.06 = moderate; ω^2^ > 0.14 = large (Field, 2013).

#### Assessment of multisensory task performance accuracy in context with other measures

To estimate the accuracy (number of errors made by each participant) in the multisensory task, the error rate percentages were calculated for each stimulus type (AS, VS, and AVS) and for each participant, Bayesian repeated measures ANOVA, followed by *post hoc* comparison was performed to calculate the group differences in MRT accuracy.

#### Bayesian correlations analyses

Bayesian correlations analyses were also performed to explore the relationships between MRT to multisensory and unisensory stimuli, degree of multisensory facilitation, visuomotor and non-motor visual perceptual processing (IT), separately for each group. The Pearson correlation coefficient (r), the Bayes Factor (BF_10_), and credible intervals (95% CI) are reported.

#### Race model and multisensory facilitation

The degree of multisensory facilitation was computed to quantify multisensory benefits on MRTs for audiovisual stimuli as compared to unisensory for each participant. The following calculation from Denervaud et al. (2020) was utilized:


RT[%]=

$$\frac{\text{faster}\,\mathrm{unisensory}\,\mathrm{MRTs}-\mathrm{multisensory}\,\mathrm{MRTs}}{\mathrm{Faster}\,\mathrm{unisensory}\,\mathrm{MRTs}}\times 100$$

In addition to this, the Race Model Inequality analysis as described in Ulrich et al. (2007) was conducted to determine whether motor responses of AVS were faster than unisensory AS and VS as discussed previously. MATLAB software (R2020a, Mathworks, Inc.), and the RMItest program were used to analyze data from the multisensory task. In this model, the cumulative density functions (CDFs) of the MRTs were calculated for individual participants, and for each stimulus condition (VS, AS, and AVS). The MRT from the two unisensory conditions were then calculated for each participant in order to predict the violations of the Race Model. We then computed the distribution of MRTs for 10 percentiles from 0.05th to 0.95th for each child, and each stimuli condition. Bayesian paired sample *t-*tests were used to compare the MRTs of the compound (auditory and visual) vs audiovisual stimuli for each percentile and each group (5−6, 7−8, and 9−10), using the default Cauchy prior width (0.707). Finally, the percentage of participants who showed faster MRTs for audiovisual stimuli than for the compound AS+VS were computed for each age group. All Bayesian hypothesis tests (ANOVA, correlations and paired sample *t-*tests) were performed in JASP 0.16.3.0 (JASP Team, 2022^2^).

## Results

### Results 1: Age-group differences in motor reaction tasks (auditory stimulus, visual stimulus, audiovisual stimulus), (SLURP) and non-motor visual perceptual processing inspection time

A series of Bayesian one-way ANOVAs were conducted to determine whether there were age-related differences in the MRT for AS, VS and AVS, SLURP, and non-motor visual perceptual processing (IT) between the three age groups. This analysis was then supported by traditional parametric ANOVA measures and reported in the Supplementary material. Table 2 presents the descriptive statistics for all dependent measures.

**TABLE 2:** Descriptive statistics for auditory RT, visual RT and audiovisual RT, visuomotor, and visual perceptual processing by age groups.

					95% Credible interval	
	Measure	Age	*M*	*SD*	Lower	Upper
MRT of Multisensory Task	Auditory RT (ms)	5−6 years 7−8 years 9−10 years	955.765 893.720 719.278	148.061 98.229 82.780	891.739 853.173 678.112	1019.792 934.267 760.443
	Visual RT (ms)	5−6 years 7−8 years 9−10 years	1024.587 915.160 746.333	109.109 111.486 52.824	977.405 869.141 720.000	1071.769 961.179 772.602
	Audiovisual RT (ms)	5−6 years 7−8 years 9−10 years	915.174 848.080 673.000	98.299 97.388 66.436	872.666 807.880 639.962	957.682 888.280 706.038
Visuo-motor	SLURP (seconds)	5−6 years 7−8 years 9−10 years	82.182 67.900 52.222	19.358 17.947 8.257	73.599 59.501 48.116	90.765 79.299 56.328
Non-motor (IT)	Inspection Time (ms)	5−6 years 7−8 years 9−10 years	109.202 68.442 57.974	49.774 24.302 19.178	83.611 58.411 48.113	134.793 78.474 67.834

**FIGURE 5:**
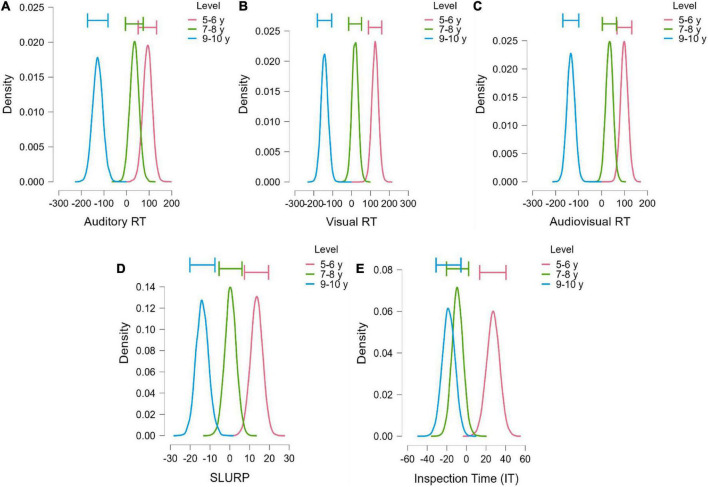
The model-averaged posterior distribution (horizontal bars show the 95% credible intervals around the median) in MRTs across three conditions **(A)** auditory (AS), **(B)** visual (VS) and **(C)** audiovisual (AVS), **(D)** visuomotor processing (SLURP), and **(E)** visual perceptual processing (IT).

Bayesian one-way ANOVA of MRT for unisensory and multisensory processing speed revealed decisive differences between the three age groups in *Auditory RT* that supported the alternative hypothesis (BF_10_ = 247045.85,ω^2^ = 0.39). *Post hoc* comparisons showed that there were decisive differences between the 9−10 group and 5−6, 7−8 group, while there was no evidence of differences between 7−8 and 5−6 group (Figure 5A and Table 3A). *Visual RT* also showed decisive differences between the three age groups that supported the alternative hypothesis (BF_10_ = 1.365e+9, ω^2^ = 0.54). *Post hoc* comparisons showed that these differences were caused by the 9−10 group being decisively greater than 5−6 and 7−8 group. There was also a strong difference between the 7−8 and 5−6 groups (Figure 5B and Table 3B). *Audiovisual RT* also indicated decisive evidence of the alternative hypothesis (BF_10_ = 3.782e+8, ω^2^ = 0.52), that was due to 9−10 group performing decisively better than 5−6 and 7−8 group. There were only anecdotal level differences between 5−6 and 7−8 group (Figure 5C and Table 3C).

**TABLE 3:** Bayesian *post hoc* comparisons for auditory RT, visual RT and audiovisual RT, visuomotor, and visual perceptual processing.

		Prior odds	Posterior odds	BF_10, U_	error %
**(A) Auditory RT**					
5−6 years	7−8 years	0.587	0.558	0.950	0.007
	9−10 years	0.587	16098.634	27406.546	2.981e−10
7−8 years	9−10 years	0.587	22863.582	38923.290	1.893e−10
**(B) Visual RT**					
5−6 years	7−8 years	0.587	14.949	25.449	5.197e−7
	9−10 years	0.587	9.516e+8	1.620e+9	1.663e−13
7−8 years	9−10 years	0.587	13503.242	22988.113	2.496e−10
**(C) Audiovisual RT**					
5−6 years	7−8 years	0.587	1.569	2.671	0.009
	9−10 years	0.587	6.724e+7	1.145e+8	1.987e−12
7−8 years	9−10 years	0.587	92451.871	157391.396	1.136e−10
**(D) SLURP**					
5−6 years	7−8 years	0.587	1.870	3.183	0.009
	9−10 years	0.587	16998.465	28938.432	3.458e−10
7−8 years	9−10 years	0.587	11.914	20.283	1.479e−6
**(E) Inspection Time (IT)**					
5−6 years	7−8 years	0.587	17.562	29.897	7.358e−7
	9−10 years	0.587	39.489	67.226	4.781e−7
7−8 years	9−10 years	0.587	0.431	0.734	0.006

The posterior odds have been corrected for multiple comparisons by fixing to 0.5 the prior probability that the null hypothesis holds across all comparisons (Westfall et al., 1997). Individual comparisons are based on the default *t*-test with a Cauchy [0, *r* = 1/sqrt(2)] prior. The “U” in the Bayes factor denotes that it is uncorrected.

*Visuo-motor processing (SLURP)* also demonstrated a decisive difference between groups (BF_10_ = 8537.115,ω^2^= 0.34) in favor of the alternative hypothesis. *Post hoc* comparisons showed that there were strong to decisive differences between the 9−10 and the 5−6 and 7−8 groups. Moderate evidence of differences between 7−8 and 5−6 groups was also found (Figure 5D and Table 3D).

By comparison, *non-motor Inspection Time* for simple visual perceptual processing demonstrated decisive differences between groups (BF_10_ = 452.346, ω^2^ = 0.27). *Post hoc* comparisons showed that strong to very strong differences existed between the 5−6 group and 9−10, 7−8 group, though no differences were found between 7−8 and 9−10 groups (Figure 5E and Table 3E). The lack of difference between the older groups supported the null hypothesis, and indicated that the youngest group (5−6 years) required a significantly longer exposure time to identify the visual stimuli in comparison to other age groups.

### Results 2: Assessment of errors in multisensory task performance

**FIGURE 6:**
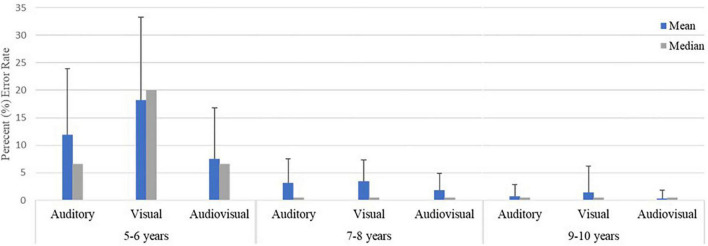
Median and mean (±SD) percentage error (%) for the three trial conditions auditory (AS), visual (VS), and audiovisual (AVS) stimuli.

The response accuracy rate to the stimuli of the multisensory task (AS, VS, and AVS) was computed and analyzed for each participant. As presented in Figure 6, the percent error rate for AS, VS, and AVS was low in both older groups (7−8 and 9−10), with the median percent error score being 0, and indicating total accuracy in responding to AS, VS, and AVS stimuli. By contrast, the youngest (5−6) age group showed a higher error rate for VS than AS and AVS trials, with a median percent error score of 20, 6.6, and 6.6, respectively. Inspection of Figure 6 shows that, on average, children displayed the greatest level of accuracy with the AVS task where the percent of errors decreased for all age groups. For the 5−6 age group, Bayesian repeated measures test showed that there was strong evidence of a difference in percent error for detection of AS, VS, and AVS in favor of the alternative hypothesis, (BF_10_ = 17.89). *Post hoc* comparison revealed strong evidence in the error rate between VS vs. AVS (BF_10,U_ = 21.61), and anecdotal evidence between VS vs. AS (BF_10,U_ = 1,26), showing that young children (5−6 age group) made more errors with VS than AS and AVS, with no evidence of differences between AS vs AVS (BF_10,U_ = 0.79). However, there was no evidence of differences in error rates for AS, VS and AVS amongst the 7−8 age group (BF_10_ = 0.39) and the 9−10 age group (BF_10_ = 0.23).

### Results 3: Relationships among motor reaction tasks to auditory stimulus, visual stimulus, audiovisual stimulus, multisensory facilitation, SLURP and inspection time task

To explore the relationships between MRT to AS, VS, and AVS, multisensory facilitation, SLURP and non-motor visual perception processing (IT), Bayesian correlations were performed separately for each age group (5−6, 7−8, and 9−10 years). For the 5−6 group, results revealed moderate evidence of the correlations in favor of the alternative hypothesis between MRT AVS, and SLURP, MRT facilitation and SLURP, indicating that faster multisensory MRT was associated with the timed motor component of SLURP. Further, there was moderate to decisive evidence of correlations between MRT to AS, VS and AVS in favor of the alternative hypothesis (Table 4). For the 7−8 group, there was decisive evidence of correlations between MRT to AS, VS, and AVS in favor of the alternative hypothesis (Table 5). For the 9−10 group, a pattern of strengthening correlations from strong to decisive was found between MRT to AS, VS, AVS, and SLURP in favor of the alternative hypothesis, suggesting that the faster MRT of multisensory tasks are more likely to be related to the motor component of SLURP than to the visual component. There was also moderate evidence of the association between MRT AVS and facilitation in this age group which supports the alternative hypothesis (Table 6). However, there were no statistically supported correlations in any of the three age groups between any MRT measures for the multisensory task components or SLURP or the threshold time for the non-motor IT task.

**TABLE 4:** Bayesian Pearson Correlations, 5−6 group.

		Pearson’s r	BF_10_	Lower 95% CI	Upper 95% CI
MRT AS	−MRT VS	0.496	3.933	0.089	0.731
MRT AS	−MRT AVS	0.765***	1226.169	0.473	0.885
MRT AS	−Facilitation	0.377	1.142	−0.044	0.657
MRT AS	−SLURP	0.251	0.480	−0.181	0.579
MRT AS	−IT	−0.110	0.325	−0.521	0.361
MRT VS	−MRT AVS	0.645**	46.639	0.285	0.819
MRT VS	−Facilitation	0.115	0.294	−0.294	0.476
MRT VS	−SLURP	0.394	1.238	−0.038	0.672
MRT VS	−IT	0.105	0.323	−0.364	0.518
MRT AVS	−Facilitation	−0.211	0.402	−0.546	0.208
MRT AVS	−SLURP	0.519	4.715	0.106	0.749
MRT AVS	−IT	−0.139	0.342	−0.541	0.337
Facilitation	−SLURP	−0.530	5.441	−0.756	−0.12
Facilitation	−IT	0.062	0.308	−0.397	0.488
SLURP	−IT	0.024	0.310	−0.438	0.473

* BF_10_ > 10, **BF_10_ > 30, ***BF_10_ > 100; MRT AS, auditory stimuli; MRT VS, visual stimuli; MRT AVS, audiovisual stimuli; Facilitation, percentage of multisensory facilitation; SLURP, visual motor skills; IT, Inspection Time.

**TABLE 5:** Bayesian Pearson Correlations, 7−8 group.

		Pearson’s r	BF_10_	Lower 95% CI	Upper 95% CI
MRT AS	−MRT VS	0.699***	312.726	0.386	0.845
MRT AS	−MRT AVS	0.685***	210.376	0.365	0.837
MRT AS	−Facilitation	0.211	0.403	−0.191	0.534
MRT AS	−SLURP	−0.098	0.300	−0.486	0.336
MRT AS	−IT	0.168	0.336	−0.231	0.503
MRT VS	−MRT AVS	0.866***	613859.466	0.682	0.936
MRT VS	−Facilitation	−0.045	0.254	−0.410	0.337
MRT VS	−SLURP	−0.169	0.351	−0.536	0.276
MRT VS	−IT	0.165	0.333	−0.233	0.501
MRT AVS	−Facilitation	−0.416	1.871	−0.673	−0.017
MRT AVS	−SLURP	−0.148	0.332	−0.521	0.294
MRT AVS	−IT	0.131	0.299	−0.264	0.476
Facilitation	−SLURP	−0.017	0.277	−0.426	0.400
Facilitation	−IT	−0.021	0.249	−0.390	0.357
SLURP	−IT	−0.176	0.358	−0.541	0.270

*BF_10_ > 10, **BF_10_ > 30, ***BF_10_ > 100; MRT AS, auditory stimuli; MRT VS, visual stimuli; MRT AVS, audiovisual stimuli; Facilitation, percentage of multisensory facilitation; SLURP, visual motor skills; IT, Inspection Time.

**TABLE 6:** Bayesian Pearson Correlations, 9−10 group.

		Pearson’s r	BF_10_	Lower 95% CI	Upper 95% CI
MRT AS	−MRT VS	0.758***	132.296	0.396	0.893
MRT AS	−MRT AVS	0.418	1.164	−0.064	0.708
MRT AS	−Facilitation	0.402	1.037	−0.081	0.699
MRT AS	−SLURP	0.6	7.222	0.153	0.811
MRT AS	−IT	0.448	1.353	−0.048	0.731
MRT VS	−MRT AVS	0.698**	35.5	0.296	0.863
MRT VS	−Facilitation	−0.017	0.292	−0.445	0.421
MRT VS	−SLURP	0.742**	91.346	0.369	0.886
MRT VS	−IT	0.35	0.723	−0.149	0.674
MRT AVS	−Facilitation	−0.598	7.041	−0.81	−0.151
MRT AVS	−SLURP	0.488	2.074	0.014	0.749
MRT AVS	−IT	0.249	0.46	−0.244	0.612
Facilitation	−SLURP	0.176	0.366	−0.293	0.557
Facilitation	−IT	0.090	0.317	−0.376	0.508
SLURP	−IT	0.302	0.569	−0.196	0.645

*BF_10_ > 10, **BF_10_ > 30, ***BF_10_ > 100; MRT AS, auditory stimuli; MRT VS, visual stimuli; MRT AVS, audiovisual stimuli; Facilitation, percentage of multisensory facilitation; SLURP, visual motor skills; IT, Inspection Time.

### Results 4: Race model comparisons of motor reaction times to unisensory and multisensory stimuli

As Bayesian ANOVAs analysis showed that MRTs of multisensory task (visual, auditory, and audiovisual) were significantly different across age groups, a Race Model analysis was performed to investigate the facilitation of multisensory integration across the three groups. Race Model analyses were performed for each age group as a whole before separating data by individual participant (see Table 7). For the 9−10 group, Bayesian *t*-tests indicated that there was strong evidence that the AVS CDF violated the assumption of the model from the 15th to 25th percentile (BF_10_ = 27.8) and there was also anecdotal evidence at the 15th percentile in favor of the alternative hypothesis, indicating AVS CDF was faster than the AS+VS CDF in this group. However, there was decisive evidence at the 95th percentile in favor of the null hypothesis, indicating the AVS CDF was slower than the AS+VS CDF at the 95th percentile. In contrast, for the 7−8 and 5−6 groups, there was no evidence for violation of inequality, and all probabilities at 45th and above showed anecdotal to decisive evidence in favor of the null hypothesis, indicating AVS CDF was slower than the AS+VS CDF, suggesting no evidence for multisensory facilitation.

**TABLE 7:** Race model inequality analysis results by three age groups using Bayesian paired samples *t*-test to compare mean MRTs for AVS and the compound AS+VS.

Probability	5−6 years				7−8 years				9−10 years			
	Mean MRTs for AVS	AS+VS (CDFs)	*BF*_10_	*Error %*	Mean MRTs for AVS	AS+VS (CDFs)	*BF*_10_	*Error %*	Mean MRTs for AVS	Mean AS+VS (CDFs)	*BF*_10_	*Error %*
**0.05**	735.98	720.02	0.442	0.023	686.63	697.67	0.219	0.026	564.31	580.83	0.650	0.022
**0.15**	769.41	774.26	0.251	0.020	726.93	741.88	0.326	0.027	595.16	609.09	***27.809***	1.327e−5
**0.25**	798.78	799.40	0.230	0.019	753.27	762.59	0.215	0.026	610.45	624.19	***27.826***	1.248e−5
**0.35**	824.31	818.95	0.233	0.019	781.81	778.37	0.577	0.026	625.87	638.14	***1.93***	1.785e−6
**0.45**	854.78	835.40	0.550	0.024	803.92	792.52	1.796	1.728e−6	638.19	650.59	0.917	0.023
**0.55**	881.47	851.49	2.760	8.383e−7	826.73	808.19	17.664	5.928e−8	654.88	659.28	0.316	0.017
**0.65**	914.11	870.20	4.488	2.652e−7	853.48	823.64	1104.242	9.864e−9	669.94	668.80	0.247	0.015
**0.75**	947.98	887.32	32.153	7.188e−4	879.07	837.82	6261.545	3.117e−9	694.03	680.32	0.963	0.024
**0.85**	1004.78	902.20	356.616	3.942e−8	919.73	850.95	74881.027	2.066e−7	723.73	691.85	19.165	0.002
**0.95**	1095.37	918.30	23260.110	1.334e−8	995.98	865.55	399463.825	1.185e−9	799.25	702.34	36795.6	2.953e−8

The bold and italic values indicate evidence in favor of the alternative hypothesis between MRTs for AVS and the compound AS+VS.

**FIGURE 7:**
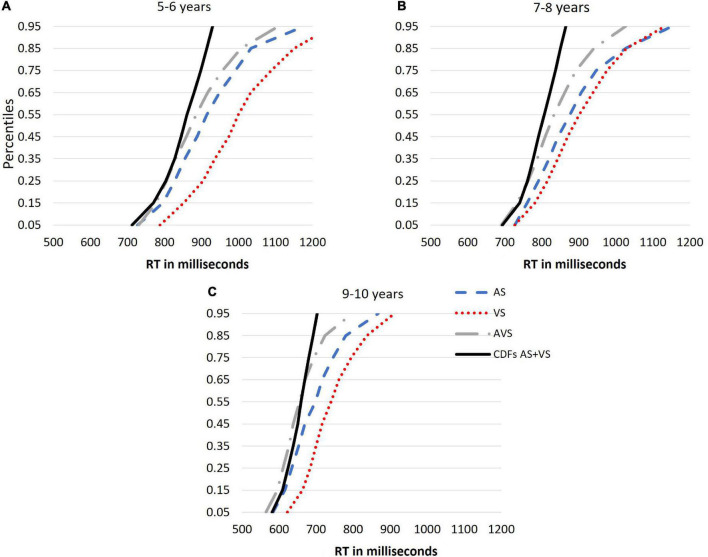
**(A–C)** Cumulative density functions (CDFs) for each individual’s auditory stimuli (AS; blue line), visual stimuli (VS; red line), audiovisual stimuli (AVS; gray line) and the combined CDF AS+VS (the black line), CDFs were computed by calculating the probability of MRTs from 0.05 to 0.95 in intervals of 0.1 (*y*-axis represent the probability).

To better understand the differences in multisensory integration across ages, the same analysis was run using *individual data* for the three age groups and similar results were obtained. The race model’s prediction analysis for the 9−10 group showed that there was evidence that AS+VS CDF was to the right of AVS for all probability values for the 45th percentile and below, except for the 5th percentile (see Figure 7C). In contrast, for the 7−8 and 5−6 groups, there was no evidence of violations (see Figures 7A,B). Observation of development in the motor speed for all three conditions AS, VS and AVS with age are apparent as in Figure 7.

**FIGURE 8:**
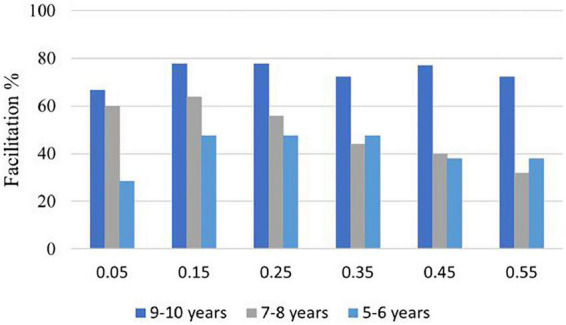
The percentage of participants in each group who show faster MRTs for AVS than AS+VS CDF predicted by the race- model at each of the six percentiles.

Inspection of individual participant responses showed that 83% (15 out 18) in the 9−10 group, 60% (15 out of 25) in the 7−8 group, and 42% (9 out of 21) in the 5−6 group showed MRTs for AVS faster than the AS+VS for at least two consecutive values at the 0.35th and below. The percentage of participants in each age group at each of the six probabilities that showed faster MRTs for AVS than AS+VS is presented in Figure 8. The six percentiles were chosen because violations of the race-model inequality were anticipated to occur (Ostrolenk et al., 2019; Ainsworth et al., 2021). Individual participants’ data and additional analyses can be found in the Supplementary material.

## Discussion

The aim of the current study was to examine motor development using audiovisual and visuomotor tasks to investigate whether such maturation of motor functions contributed more significantly to multisensory facilitation than sensory visual development and processing in three groups of early school-aged children (5−6, 7−8, and 9−10 years old). The most important findings of this study using both traditional and Bayesian analyses were significant and moderate to decisive evidence suggesting that age-related reductions in all speeded motor reaction time measures of multisensory facilitation for children aged 5−10, were primarily due to decrease in timed motor responses rather than due to increase in rate of visual sensory processing with age. We found no evidence of correlations between visual-only processing measures and multisensory facilitation further suggesting that the increase in MRT for detection of multisensory stimuli was not a function of increase in rate of visual processing *per se*. Our analyses also showed that despite faster motor responses being seen on the audiovisual and visuomotor tasks, multisensory facilitation of MRTs was observed only in the oldest group of children, consistent with race-model predictions and previous literature (Barutchu et al., 2009, 2010; Brandwein et al., 2011).

### Age and performance on measures of motor reaction times and non-motor visual perceptual processing

#### Age-related differences in multisensory and visuomotor tasks

As expected, there was moderate to decisive evidence for age-group differences in MRT on our multisensory and visuo-motor processing tasks, but not for the simple motor-free threshold measure of visual perception. Improvements in age related motor reaction time to detection of both unisensory and multisensory stimuli by school age children are in line with previous findings (Barutchu et al., 2009; Cameron et al., 2012; Square et al., 2014; Nardini et al., 2016; Alghamdi et al., 2021). Furthermore, our findings are also consistent with previous studies demonstrating age-related improvement in visual-motor integration reflected by faster sensory processing speed and fewer errors, in children until the age of 10, after which performance stabilizes, when using a variety of tasks such as the Slurp Eye-Hand Coordination Test (Junghans and Khuu, 2019), and the Purdue Pegboard Test (Gardner and Broman, 1979). Indeed, Lyzohub et al. (2019) have suggested that age related increase in speed of motor reaction tasks by children aged 7–8 and late adolescence could be due to the morphological and functional maturation in the neural networks of motor and cognitive brain systems around the precentral area of the cerebral cortex and intercentral cortices as well as the improvement of the neuromuscular system.

#### Age-related differences in IT task

Although there was Bayesian evidence to suggest age-group differences in the threshold IT task in the current study, IT performance was only different for children aged 5 to 6 but not children aged 7 and above, suggesting that the threshold for IT had begun to plateau around 5/6-years-old. The decreasing time required to successfully identify the IT task stimuli is likely to be associated with retinal development where the fovea reaches adult photoreceptor density and differentiation by approximately 5 years (Hendrickson et al., 2012). The slower rate of identification in younger children may be attributable to physiological conduction rates and latency of retinal ganglion magnocellular and parvocellular projection time from the eye to primary visual cortex, that does not reach maturity until late childhood or early adolescence (Klistorner et al., 1997; Crewther et al., 1999). Equally importantly is the fact that the youngest group of 5−6 year old, children had only recently started school and often show limited receptive language skills leading to inability to attend to and understand unusual and more complex instructions, suggesting that our results showing significant differences in the 5 to 6 age group for the IT threshold, may also be partly due to underdeveloped visual and cognitive abilities.

#### Race model analysis

Although our age group differences using traditional and Bayesian analyses are in line with previous studies and support our motoric development hypotheses, tests of multisensory facilitation-driven race model violations may still have theoretical validity to show the facilitation of multisensory integration (Ulrich et al., 2007; Downing et al., 2015; Crosse et al., 2019). Results from the current study for traditionally defined multisensory facilitation of MRT are also consistent with those found in a study conducted by Brandwein et al. (2011) that used an AV simple reaction time task to show that multisensory facilitation is still immature at around 8 years of age, but reaches mature levels later in childhood. Indeed, in the current study children in the 5−6 and 7−8 year old groups did not violate the race model prediction of inequality, though they showed faster motor responses. A possible explanation for this is the greater number of errors in these two younger groups, i.e., a decrease in MRT accuracy (Miller, 2004), as well as the variation in performance across participants (Ulrich and Giray, 1986). Barutchu et al. (2009), using a similar multisensory task to that of the current investigation, did not observe the consistent increases in multisensory facilitation we saw for children aged 6 to 11 years, with no violation being particularly apparent in two groups of children aged 6 and 10−11 years old. The race model violation we observed in children aged 9 to 10 years suggests that in many cases there is a trend in multisensory facilitation that continues into late childhood and adolescence; (Brandwein et al., 2011), with little multisensory integration occurring before 8 years of age (Gori et al., 2008, 2021; Nardini et al., 2008; Barutchu et al., 2009, 2010). This age is similar to the time when higher order visually driven cognitive processes such as sustained attention, vocabulary (Pickering et al., in press) and decision making have also begun to develop and mature (Cotton et al., 2005), similar to motor processing that continues to improve till late adolescence. Importantly our results are consistent with the electrophysiological findings of Brandwein et al. (2011) showing that the cortical regions underlying multisensory processes continue to develop as a function of age, and brain imaging investigations showing continuing development through adolescence of higher visual areas that receive Magnocellular driven dorsal visual stream information (see Klaver et al., 2011 for a review).

### Audiovisual, visuo-motor and visual perceptual processing and their relationship with multisensory facilitation

The hypothesis regarding a progressive age related decrease in MRT to multisensory and unisensory stimuli in school age children was supported by our Bayesian analyses that indicated moderate to decisive evidence for correlations between MRT to AS, VS, AVS, and SLURP in children aged 9 to 10, and is consistent with past studies that have demonstrated faster performance on the MRT measures with age (Barutchu et al., 2020; Denervaud et al., 2020). The current study also demonstrated that the level of multisensory facilitation of motor responses correlated with visuomotor (SLURP) task performance and MRT to AVS, indicating that the motor system in children no older than 10 years, is an important contributor to multisensory integration. Interestingly, there were no significant correlations between the threshold IT and any measure of MRT (audiovisual and visuo-motor) tasks and multisensory facilitation for all our groups indicating that maturation of the sensory visual processing does not contribute as significantly to multisensory facilitation as does motor maturation. This is in line with previous results by Alghamdi et al. (2021) in children aged 5 to 7, and Ebaid et al. (2017) in adults, who investigated the associations between processing speed with motor components tasks and simple visual information processing as measured with the IT task, and also found no significant correlations between motor tasks and IT.

## Limitations

The strength of this study was the use of traditional statistics with null hypothesis and Bayesian probability statistics. Furthermore, we have included a Race Model analysis to determine the age related changes in performance of school age children. However, there are several limitations to this study. First, a major limitation of our findings is that we did not independently assess non-motor auditory threshold detection times. However, the overarching aim of our experiment was to focus on the threshold time for visual recognition as both past research and the current study demonstrate that MRT for visual-motor detection is always greater than MRT for auditory-motor detection in children and adults (see Barutchu et al., 2009; Downing et al., 2015). Future studies may benefit from including both visual and auditory threshold detection tasks without any motor component. It may also be useful for future research to use other robust measures of motor reaction times such as that for eye movements, or familiar objects/sound identification rather than a non-specific Gaussian stimulus, as developmental literature in this area remains relatively rare. Furthermore, sample sizes with fewer participants in the 9−10 age group compared to the other groups was a minor limitation in the current study, thus future research should aim to obtain samples of equal sizes in the various age groups to ensure the strength of analyses and results.

## Conclusion and future directions

To our knowledge, this study is the first to measure motor speed on multisensory processing tasks (i.e., audiovisual and visuomotor tasks) in children, and to compare these measures to performance on simple threshold development of visual object identification, that was quantified using a simple non-motor Inspection time task. Our findings indicate that motor RT contributed more to threshold times of age-related multisensory integration up to 10 years than improvement in visual detection alone when measured with the IT task.

In summary, the present study used traditional and Bayesian analyses to provide a novel perspective on motor function with age and demonstrate evidence that motor responses *per se* contribute significantly to multisensory facilitation in older primary school-aged children. Furthermore, our results confirm that MRTs to multisensory stimuli continue to develop throughout childhood as the oldest children showed enhanced multisensory integration. Although prior studies reported a link between multisensory integration and the performance of cognitive development in younger and older adults (Fahey et al., 2018; Murray et al., 2018), further research is needed to investigate the contributions of cognitive tasks such as IQ, working memory to the motor development of multisensory integration in children. Results from the current study also indicate that multisensory processing tasks could be a useful tool for the assessment of sensory and motor development in a public health screening in school-aged children. Given that multisensory processing has been demonstrated to be impaired in some realms of research such as Autism (Stevenson et al., 2017; Ainsworth et al., 2021), as well as in dyslexia (Harrar et al., 2014), it would be useful to apply the current findings to the neurodevelopmental populations.

## Data availability statement

The original contributions presented in the study are included in the article/Supplementary material, further inquiries can be directed to the corresponding author.

## Ethics statement

The studies involving human participants were reviewed and approved by the La Trobe University Human Ethics Committee, the Victorian Department of Education Human Ethics Committee, and the Victorian Catholic Schools Ethics Committee. Written informed consent to participate in this study was provided by the participants’ legal guardian/next of kin.

## Author contributions

AA and SC designed and developed the study content. AA and MM conducted the data analysis. All authors contributed to the Interpretation. All authors made substantial contributions to the writing and developed the first and final version of the manuscript.

## Footnotes

1
http://www.vpixx.com/

2
http://www.jasp-stats.org/

## References

[B1] Ainsworth K. Ostrolenk A. Irion C. Bertone A. (2021). Reduced multisensory facilitation exists at different periods of development in autism. *Cortex* 134 195–206. 10.1016/j.cortex.2020.09.031 33291045

[B2] Alghamdi R. Murphy M. Goharpey N. Crewther S. (2021). The age-related changes in speed of visual perception, visual verbal and visuomotor performance, and nonverbal intelligence during early school years. *Front. Hum. Neurosci.* 15:667612. 10.3389/fnhum.2021.667612 34483862 PMC8416250

[B3] Alsius A. Navarra J. Campbell R. Soto-Faraco S. (2005). Audiovisual integration of speech falters under high attention demands. *Curr. Biol.* 15 839–843. 10.1016/j.cub.2005.03.046 15886102

[B4] Andersen R. A. Snyder L. H. Bradley D. C. Xing J. (1997). Multimodal representation of space in the posterior parietal cortex and its use in planning movements. *Annu. Rev. Neurosci.* 20 303–330. 10.1146/annurev.neuro.20.1.303 9056716

[B5] Barutchu A. Spence C. (2020). An experimenter’s influence on motor enhancements: The effects of letter congruency and sensory switch-costs on multisensory integration. *Front. Psychol.* 11:588343. 10.3389/fpsyg.2020.588343 33335500 PMC7736551

[B6] Barutchu A. Crewther D. P. Crewther S. G. (2009). The race that precedes coactivation: development of multisensory facilitation in children. *Dev. Sci.* 12 464–473. 10.1111/j.1467-7687.2008.00782.x 19371371

[B7] Barutchu A. Fifer J. M. Shivdasani M. N. Crewther S. G. Paolini A. G. (2020). The interplay between multisensory associative learning and IQ in children. *Child Dev.* 91 620–637. 10.1111/cdev.13210 30620403

[B8] Barutchu A. Toohey S. Shivdasani M. N. Fifer J. M. Crewther S. G. Grayden D. B. (2019). Multisensory perception and attention in school-age children. *J. Exp. Child Psychol.* 180 141–155. 10.1016/j.jecp.2018.11.021 30655099

[B9] Barutchu A. Spence C. Humphreys G. W. (2018). Multisensory enhancement elicited by unconscious visual stimuli. *Exp. Brain Res.* 236 409–417. 10.1007/s00221-017-5140-z 29197998 PMC5809521

[B10] Barutchu A. Danaher J. Crewther S. G. Innes-Brown H. Shivdasani M. N. Paolini A. G. (2010). Audiovisual integration in noise by children and adults. *J. Exp. Child Psychol.* 105 38–50. 10.1016/j.jecp.2009.08.005 19822327

[B11] Botta F. Santangelo V. Raffone A. Sanabria D. Lupiáñez J. Belardinelli M. O. (2011). Multisensory integration affects visuo-spatial working memory. *J. Exp. Psychol.* 37:1099. 10.1037/a0023513 21553989

[B12] Brandwein A. B. Foxe J. J. Russo N. N. Altschuler T. S. Gomes H. Molholm S. (2011). The development of audiovisual multisensory integration across childhood and early adolescence: a high-density electrical mapping study. *Cerebr. Cortex* 21 1042–1055. 10.1093/cercor/bhq170 20847153 PMC3077428

[B13] Brown A. C. Crewther D. P. (2017). Autistic children show a surprising relationship between global visual perception, non-verbal intelligence and visual parvocellular function, not seen in typically developing children. *Front. Hum. Neurosci.* 11:239. 10.3389/fnhum.2017.00239 28553216 PMC5425824

[B14] Cameron C. E. Brock L. L. Murrah W. M. Bell L. H. Worzalla S. L. Grissmer D. (2012). Fine motor skills and executive function both contribute to kindergarten achievement. *Child Dev.* 83 1229–1244. 10.1111/j.1467-8624.2012.01768.x 22537276 PMC3399936

[B15] Cohen J. (1992). A power primer. *Psychol. Bull.* 112:155. 10.1037/0033-2909.112.1.155 19565683

[B16] Corbetta M. Shulman G. L. (2002). Control of goal-directed and stimulus-driven attention in the brain. *Nat. Rev. Neurosci.* 3 201–215. 10.1038/nrn755 11994752

[B17] Corman L. Budoff M. (1974). Factor structures of retarded and nonretarded children on Raven’s Progressive Matrices. *Educ. Psychol. Meas.* 34 407–412. 10.1177/001316447403400226

[B18] Cotton S. M. Kiely P. M. Crewther D. P. Thomson B. Laycock R. Crewther S. G. (2005). A normative and reliability study for the Raven’s Coloured Progressive Matrices for primary school aged children from Victoria, Australia. *Personal. Individ. Diff.* 39 647–659. 10.1016/j.paid.2005.02.015

[B19] Couth S. Gowen E. Poliakoff E. (2018). Using race model violation to explore multisensory responses in older adults: Enhanced multisensory integration or slower unisensory processing? *Multisens. Res.* 31 151–174. 10.1163/22134808-00002588 31264629

[B20] Crewther S. G. Crewther D. P. Klistorner A. Kiely P. M. (1999). Development of the magnocellular VEP in children: implications for reading disability. *Electroencephalogr. Clin. Neurophysiol.* 49 123–128. 10533097

[B21] Crosse M. J. Foxe J. J. Tarrit K. Freedman E. G. Molholm S. (2022). Resolution of impaired multisensory processing in autism and the cost of switching sensory modality. *Commun. Biol*. 5 1–17. 10.1038/s42003-022-03519-1 35773473 PMC9246932

[B22] Crosse M. J. Foxe J. J. Molholm S. (2019). Developmental recovery of impaired multisensory processing in autism and the cost of switching sensory modality. *BioRxiv* [Preprint]. 10.1101/565333 PMC9246932 35773473

[B23] Denervaud S. Gentaz E. Matusz P. J. Murray M. M. (2020). Multisensory gains in simple detection predict global cognition in schoolchildren. *Sci. Rep.* 10 1–11. 10.1038/s41598-020-58329-4 32019951 PMC7000735

[B24] Downing H. C. Barutchu A. Crewther S. G. (2015). Developmental trends in the facilitation of multisensory objects with distractors [Original Research]. *Front. Psychol.* 5:1559. 10.3389/fpsyg.2014.01559 25653630 PMC4298743

[B25] Driver J. Noesselt T. (2008). Multisensory interplay reveals crossmodal influences on ‘sensory-specific’brain regions, neural responses, and judgments. *Neuron* 57 11–23. 10.1016/j.neuron.2007.12.013 18184561 PMC2427054

[B26] Ebaid D. Crewther S. G. MacCalman K. Brown A. Crewther D. P. (2017). Cognitive processing speed across the lifespan: beyond the influence of motor speed. *Front. Aging Neurosci.* 9:62. 10.3389/fnagi.2017.00062 28381999 PMC5360696

[B27] Fahey S. Charette L. Francis C. Zheng Z. (2018). Multisensory integration of signals for bodily self-awareness requires minimal cognitive effort. *Can. J. Exp. Psychol.* 72:244. 10.1037/cep0000152 30124314

[B28] Fairhall S. Macaluso E. (2009). Spatial attention can modulate audiovisual integration at multiple cortical and subcortical sites. *Eur. J. Neurosci.* 29 1247–1257. 10.1111/j.1460-9568.2009.06688.x 19302160

[B29] Faul F. Erdfelder E. Lang A.-G. Buchner A. (2007). G* Power 3: a flexible statistical power analysis program for the social, behavioral, and biomedical sciences. *Behav. Res. Methods* 39 175–191. 10.3758/BF03193146 17695343

[B30] Field A. (2013). *Discovering Statistics Using IBM SPSS Statistics.* Thousand Oaks, CA: Sage.

[B31] Forster B. Cavina-Pratesi C. Aglioti S. M. Berlucchi G. (2002). Redundant target effect and intersensory facilitation from visual-tactile interactions in simple reaction time. *Exp. Brain Res.* 143 480–487. 10.1007/s00221-002-1017-9 11914794

[B32] Froesel M. Cappe C. Hamed S. B. (2021). A multisensory perspective onto primate pulvinar functions. *Neurosci. Biobehav. Rev.* 125 231–243. 10.1016/j.neubiorev.2021.02.043 33662442

[B33] Gardner R. A. Broman M. (1979). The purdue pegboard: normative data on 1334 school children. *J. Clin. Child Adolesc. Psychol.* 8 156–162. 10.1080/15374417909532912

[B34] Giray M. Ulrich R. (1993). Motor coactivation revealed by response force in divided and focused attention. *J. Exp. Psychol.* 19:1278. 10.1037//0096-1523.19.6.1278 8294892

[B35] Gliem J. A. Gliem R. R. (2003). “Calculating, interpreting, and reporting Cronbach’s alpha reliability coefficient for Likert-type scales,” in *Proceedings of the 2003 Midwest Research to Practice Conference in Adult, Continuing, and Community Education, Columbus*, Genoa, 82–88. 10.1016/j.encep.2016.05.011

[B36] Goharpey N. Crewther D. P. Crewther S. G. (2013). Problem solving ability in children with intellectual disability as measured by the Raven’s colored progressive matrices. *Res. Dev. Disabil.* 34 4366–4374. 10.1016/j.ridd.2013.09.013 24139715

[B37] Gori M. Campus C. Cappagli G. (2021). Late development of audio-visual integration in the vertical plane. *Curr. Res. Behav. Sci.* 2:100043. 10.1016/j.crbeha.2021.100043

[B38] Gori M. Del Viva M. Sandini G. Burr D. C. (2008). Young children do not integrate visual and haptic form information. *Curr. Biol.* 18 694–698. 10.1016/j.cub.2008.04.036 18450446

[B39] Harrar V. Tammam J. Pérez-Bellido A. Pitt A. Stein J. Spence C. (2014). Multisensory integration and attention in developmental dyslexia. *Curr. Biol.* 24 531–535. 10.1016/j.cub.2014.01.029 24530067

[B40] Hendrickson A. Possin D. Vajzovic L. Toth C. A. (2012). Histologic development of the human fovea from midgestation to maturity. *Am. J. Ophthalmol.* 154 767.e2–778.e2. 10.1016/j.ajo.2012.05.007 22935600 PMC3509500

[B41] JASP Team (2022). *JASP (Version 0.16.3)[Computer software].*

[B42] Jung R. E. Haier R. J. (2007). The parieto-frontal integration theory (P-FIT) of intelligence: converging neuroimaging evidence. *Behav. Brain Sci.* 30 135–154. 10.1017/S0140525X07001185 17655784

[B43] Junghans B. M. Khuu S. K. (2019). Populations Norms for “SLURP”—An iPad App for quantification of visuomotor coordination testing [Original Research]. *Front. Neurosci.* 13:711. 10.3389/fnins.2019.00711 31354420 PMC6636550

[B44] Kiesel A. Steinhauser M. Wendt M. Falkenstein M. Jost K. Philipp A. M. (2010). Control and interference in task switching—A review. *Psychol. Bull.* 136:849. 10.1037/a0019842 20804238

[B45] Klaver P. Marcar V. Martin E. (2011). Neurodevelopment of the visual system in typically developing children. *Prog. Brain Res*. 189 113–136. 10.1016/B978-0-444-53884-0.00021-X 21489386

[B46] Klistorner A. Crewther D. Crewther S. (1997). Separate magnocellular and parvocellular contributions from temporal analysis of the multifocal VEP. *Vis. Res.* 37 2161–2169. 10.1016/S0042-6989(97)00003-5 9327064

[B47] Knopfel T. Sweeney Y. Radulescu C. I. Zabouri N. Doostdar N. Clopath C. (2019). Audio-visual experience strengthens multisensory assemblies in adult mouse visual cortex. *Nat. Commun.* 10:5684. 10.1038/s41467-019-13607-2 31831751 PMC6908602

[B48] Lakens D. (2013). Calculating and reporting effect sizes to facilitate cumulative science: a practical primer for t-tests and ANOVAs. *Front. Psychol.* 4:863. 10.3389/fpsyg.2013.00863 24324449 PMC3840331

[B49] Lee K. Junghans B. M. Ryan M. Khuu S. Suttle C. M. (2014). Development of a novel approach to the assessment of eye–hand coordination. *J. Neurosci. Methods* 228 50–56. 10.1016/j.jneumeth.2014.02.012 24657494

[B50] Liu Y. Otto T. U. (2020). The role of context in experiments and models of multisensory decision making. *J. Math. Psychol.* 96:102352. 10.1016/j.jmp.2020.102352

[B51] Lyzohub V. Chernenko N. Kozhemiako T. Palabiyik À Bezkopylna S. (2019). Age peculiarities of interaction of motor and cognitive brain systems while processing information of different modality and complexity. *Regul. Mech. Biosyst.* 10 288–294. 10.15421/021944 23636723

[B52] Miall R. Reckess G. (2002). The cerebellum and the timing of coordinated eye and hand tracking. *Brain Cogn.* 48 212–226. 10.1006/brcg.2001.1314 11812043

[B53] Miller J. (1982). Divided attention: evidence for coactivation with redundant signals. *Cogn. Psychol.* 14 247–279. 10.1016/0010-0285(82)90010-X 7083803

[B54] Miller J. (2004). Exaggerated redundancy gain in the split brain: a hemispheric coactivation account. *Cogn. Psychol.* 49 118–154. 10.1016/j.cogpsych.2003.12.003 15304369

[B55] Murray M. M. Eardley A. F. Edginton T. Oyekan R. Smyth E. Matusz P. J. (2018). Sensory dominance and multisensory integration as screening tools in aging. *Sci. Rep.* 8:8901. 10.1038/s41598-018-27288-2 29891964 PMC5995929

[B56] Nardini M. Bales J. Mareschal D. (2016). Integration of audio-visual information for spatial decisions in children and adults. *Dev. Sci.* 19 803–816. 10.1111/desc.12327 26190579

[B57] Nardini M. Jones P. Bedford R. Braddick O. (2008). Development of cue integration in human navigation. *Curr. Biol.* 18 689–693. 10.1016/j.cub.2008.04.021 18450447

[B58] Nettelbeck T. Wilson C. (2004). The Flynn effect: smarter not faster. *Intelligence* 32 85–93. 10.1016/S0160-2896(03)00060-6

[B59] Nettelbeck T. Young R. (1990). Inspection time and intelligence in 7-yr-old children: a follow-up. *Personal. Individ. Diff.* 11 1283–1289. 10.1016/0191-8869(90)90155-K

[B60] Nuzzo R. L. (2017). An introduction to bayesian data analysis for correlations. *PM R* 9 1278–1282. 10.1016/j.pmrj.2017.11.003 29274678

[B61] Olejnik S. Algina J. (2003). Generalized eta and omega squared statistics: measures of effect size for some common research designs. *Psychol. Methods* 8:434. 10.1037/1082-989X.8.4.434 14664681

[B62] Ostrolenk A. Bao V. A. Mottron L. Collignon O. Bertone A. (2019). Reduced multisensory facilitation in adolescents and adults on the autism spectrum. *Sci. Rep.* 9 1–9. 10.1038/s41598-019-48413-9 31427634 PMC6700191

[B63] Otto T. U. Mamassian P. (2012). Noise and correlations in parallel perceptual decision making. *Curr. Biol.* 22 1391–1396. 10.1016/j.cub.2012.05.031 22771043

[B64] Pecher D. Zeelenberg R. (2022). Does multisensory study benefit memory for pictures and sounds? *Cognition* 226:105181. 10.1016/j.cognition.2022.105181 35640373

[B65] Pickering H. E. Peters J. L. Crewther S. (2021). A role for visual memory in vocabulary development: a systematic review and meta-analysis. *Neuropsychol. Rev.* 10.31234/osf.io/4qyvs [Epub ahead of print]. PMC10770228 36136174

[B66] Pinto J. O. Vieira, De Melo B. B. Dores A. R. Peixoto B. Geraldo A. (2021). Narrative review of the multisensory integration tasks used with older adults: Inclusion of multisensory integration tasks into neuropsychological assessment. *Expert Rev. Neurother.* 21 657–674. 10.1080/14737175.2021.1914592 33890537

[B67] Poole D. Miles E. Gowen E. Poliakoff E. (2021). Shifting attention between modalities: Revisiting the modality-shift effect in autism. *Attent. Percept. Psychophys.* 83 2498–2509. 10.3758/s13414-021-02302-4 33939157 PMC8302542

[B68] Raab D. H. (1962). Division of psychology: statistical facilitation of simple reaction times. *Trans. N.Y. Acad. Sci.* 24 574–590. 10.1111/j.2164-0947.1962.tb01433.x 14489538

[B69] Raven J. C. Court J. H. (1990). *Coloured progressive matrices*. Oxford: Oxford Psychologists Press.

[B70] Raven J. Court J. Raven J. C. (1988). *Manual for Raven’s Progressive Matrices and Vocabulary Scales.* Oxford: Oxford Psychologists Press.

[B71] Rose S. A. Feldman J. F. Jankowski J. J. Van Rossem R. (2008). A cognitive cascade in infancy: pathways from prematurity to later mental development. *Intelligence* 36 367–378. 10.1016/j.intell.2007.07.003 19122757 PMC2504323

[B72] Sandhu R. Dyson B. J. (2013). Modality and task switching interactions using bi-modal and bivalent stimuli. *Brain Cogn.* 82 90–99. 10.1016/j.bandc.2013.02.011 23524240

[B73] Shaw L. H. Freedman E. G. Crosse M. J. Nicholas E. Chen A. M. Braiman M. S. (2020). Operating in a multisensory context: assessing the interplay between multisensory reaction time facilitation and inter-sensory task-switching effects. *Neuroscience* 436 122–135. 10.1016/j.neuroscience.2020.04.013 32325100

[B74] Square P. A. Namasivayam A. K. Bose A. Goshulak D. Hayden D. (2014). Multi-sensory treatment for children with developmental motor speech disorders. *Int. J. Lang. Commun. Disord.* 49 527–542. 10.1111/1460-6984.12083 24617702

[B75] Stein B. E. Burr D. Constantinidis C. Laurienti P. J. Alex Meredith M. Perrault T. J. Jr. (2010). Semantic confusion regarding the development of multisensory integration: a practical solution. *Eur. J. Neurosci.* 31 1713–1720. 10.1111/j.1460-9568.2010.07206.x 20584174 PMC3055172

[B76] Stein B. E. Meredith M. A. (1993). *The Merging of the Senses.* Cambridge, MA: The MIT press.

[B77] Stevenson R. A. Segers M. Ncube B. L. Black K. R. Bebko J. M. Ferber S. (2017). The cascading influence of multisensory processing on speech perception in autism. *Autism* 22 609–624. 10.1177/1362361317704413 28506185

[B78] Talsma D. Senkowski D. Soto-Faraco S. Woldorff M. G. (2010). The multifaceted interplay between attention and multisensory integration. *Trends Cogn. Sci.* 14 400–410. 10.1016/j.tics.2010.06.008 20675182 PMC3306770

[B79] Todd J. W. (1912). *Reaction to Multiple Stimuli.* Beijing: Science Press.

[B80] Ulrich R. Giray M. (1986). Separate-activation models with variable base times: testability and checking of cross-channel dependency. *Percept. Psychophys.* 39 248–254. 10.3758/BF03204931 3737352

[B81] Ulrich R. Miller J. Schroter H. (2007). Testing the race model inequality: an algorithm and computer programs. *Behav. Res. Methods* 39 291–302. 10.3758/BF03193160 17695357

[B82] Vickers D. Nettelbeck T. Willson R. (1972). Perceptual indices of performance: the measurement of ‘inspection time’and ‘noise’in the visual system. *Perception* 1 263–295. 10.1068/p010263 4680931

[B83] Westfall P. H. Johnson W. O. Utts J. M. (1997). A Bayesian perspective on the Bonferroni adjustment. *Biometrika* 84 419–427. 10.1093/biomet/84.2.419

[B84] Wetzels R. Wagenmakers E.-J. (2012). A default Bayesian hypothesis test for correlations and partial correlations. *Psychon. Bull. Rev.* 19 1057–1064. 10.3758/s13423-012-0295-x 22798023 PMC3505519

[B85] Wijesundera C. Crewther S. G. Wijeratne T. Vingrys A. J. (2022). Vision and visuomotor performance following acute ischemic stroke. *Front. Neurol.* 13:757431. 10.3389/fneur.2022.757431 35250804 PMC8889933

