# Supplementary material for: Age-related decrease in motor contribution to multisensory reaction times in primary school children
Source: Front Hum Neurosci. 2022 Sep 8;16:967081. doi: 10.3389/fnhum.2022.967081 (PMC9493199; doi:10.3389/fnhum.2022.967081)
Supplement: Supplementary file 1 [file Data_Sheet_1.PDF]

Supplementary Material

Age-Related Decrease in Multisensory Motor Reaction Times in Primary School Children

Areej A. Alhamdan [*], Melanie J. Murphy & Sheila G. Crewther

---

Results 1: Age-Group Differences in MRT (AS, VS and AVS), (SLURP) and Non-motor visual perceptual processing (IT)

The parametric ANOVA measures of MRT for unisensory and multisensory processing speed revealed significant age-group differences in Auditory RT (F (2.66) = 25.06, p < .001, η2= .43), Visual RT (F (2.66) = 43.27, *p* < .001, η2 = .56), and Audiovisual RT (F (2.64) = 47.17, *p* < .001, η2= 59). Scheffe post hoc tests showed that MRT for audiovisual RT and Visual RTdid significantly differ for all three age groups. However, Auditory RT was significantly faster only on the older group 9-10 years, where the 5-6 and 7-8 age groups did not differ (*p* = .18) (Supplementary Figure 1).

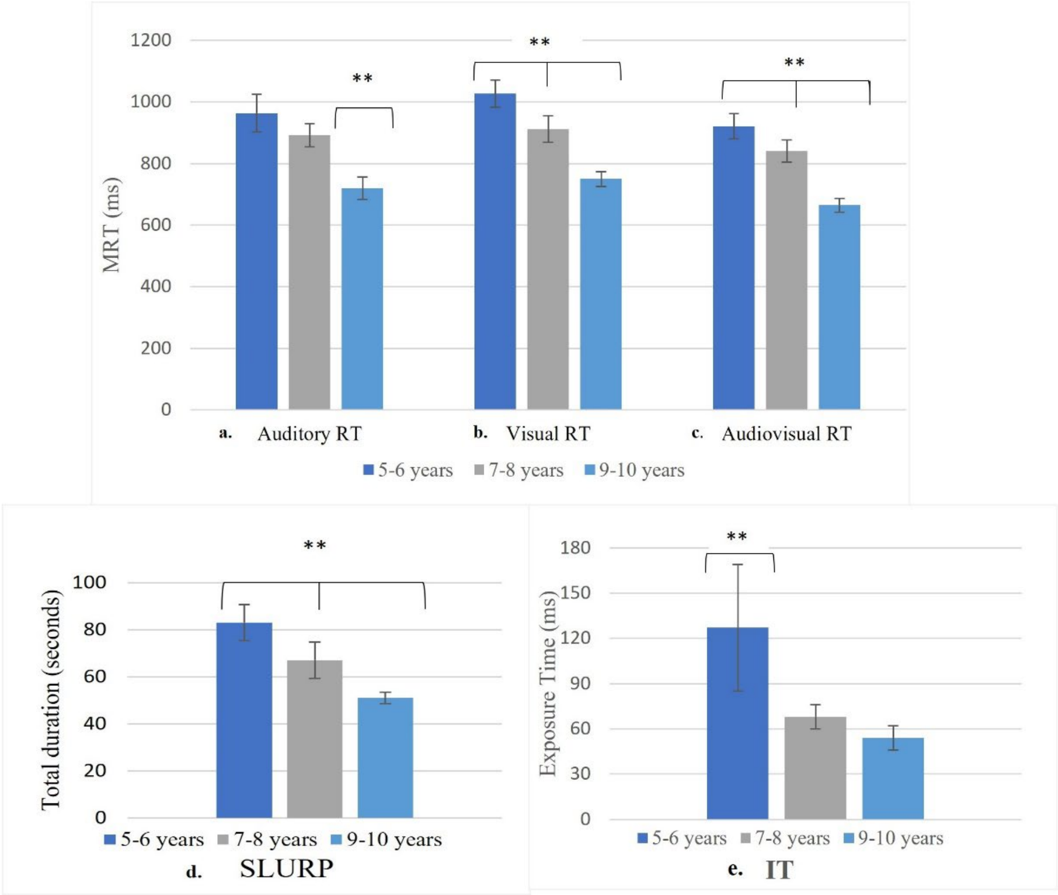

Supplementary Figure 1. Age group differences (±95% Confidence Intervals) in MRTs across three conditions (a) auditory RT, (b) visual RT and (c) audiovisual RT, (d) visuomotor processing (SLURP), and (e) visual perceptual processing (IT). **Note.** ***p* ≤ 0.01

Results 2: Assessment of Errors in Multisensory Task Performance

For the 5-6 age group, a Friedman test showed that there was a statistically significant difference in percent error for detection of AS, VS, and AVS, χ^2 (2) = 11.37, p = .003. Post hoc analysis with Wilcoxon signed-rank tests revealed a significant difference in the error rate between VS vs AS, and VS vs AVS (Z = -2.060, p = .03) and (Z = -2.575, p = .004), respectively, showing young children (5-6 age group) made more errors with VS than AS and AVS, with no significant differences between AS vs AVS (Z = -1.665, p = .09). In addition, Friedman tests showed that error rates for AS, VS and AVS did not significantly differ amongst the 7-8 age group χ^2 (2) = 2.73, p = .25, and the 9-10 age group χ^2 (2) = 1.07, p = .58.

Results 3: Relationships among MRT to AS, VS and AVS, multisensory facilitation, SLURP and IT task

Supplementary Table 1. Pearson's Correlations for the three age groups between MRTs for Inspection Time (IT), Visual (VS), Auditory (AS), Audiovisual (AVS), Multisensory Facilitation and SLURP

Measure	1	2	3	4	5	6
**1. IT**	_	-0.110	0.105	-0.139	0.062	0.024
**2. MRT AS**	0.168	_	.496*	.765**	0.377	0.251
**3. MRT VS**	0.165	.699**	_	.645**	0.115	0.394
**4. MRT AVS**	0.131	.685**	.866**	_	-0.211	.519*
**5. Facilitation**	-0.021	0.211	-0.045	-.416*	_	-.530*
**6. SLURP**	-0.176	-0.098	-0.169	-0.148	-0.017	_
**1. IT**	_	0.448	0.350	0.249	0.090	0.302
**2. MRT AS**		_	.758**	0.418	0.402	.600**
**3. MRT VS**			_	.698**	-0.016	.742**
**4. MRT AVS**				_	-.598**	.488*
**5. Facilitation**					_	0.176
**6. SLURP**						_

Note. 5- 6 years old data shaded in gray , 7-8 years old data shaded in blue , and 9-10 years old data shaded in yellow .

IT = Inspection Time; MRT AS = auditory stimuli; MRT VS = visual stimuli; MRT AVS = audiovisual stimuli; Facilitation = percentage of multisensory facilitation; SLURP= visual motor skills.

** $p \leq 0.01$, *$p \leq 0.05$.

Results 4: Race Model Comparisons of Motor Reaction Times to Unisensory and Multisensory Stimuli

Supplementary Table 2. Race Model Inequality Analysis Results by Three Age Groups. Note. * indicates *p*-value for one tailed paired sample t-test between mean MRTs for AVS and the compound AS+VS. For all t-tests results were indicated as significant when *p* after Benjamini and Hochberg correction for multiple comparisons *p*-cor was lower than 0.05.

Probability	***5-6 years***				***7-8 years***				***9-10 years***			
	**Mean MRTs for AVS**	**AS+VS (CDFs)**	***t*-value**	***p*-value**	**Mean MRTs for AVS**	**AS+VS (CDFs)**	***t*-value**	***p*-value**	**Mean MRTs for AVS**	**AS+VS (CDFs)**	***t*-value**	***p*-value**
**0.05**	735.98	720.02	-1.231	.11	686.63	697.67	0.280	.39	564.31	580.83	1.528	.07
**0.15**	769.41	774.26	-0.471	.32	726.93	741.88	0.984	.16	595.16	609.09	3.807	**.000***
**0.25**	798.78	799.40	-0.154	.43	753.27	762.59	-0.216	.41	610.45	624.19	3.805	**.000***
**0.35**	824.31	818.95	-0.226	.41	781.81	778.37	-1.514	.07	625.87	638.14	2.298	**.01***
**0.45**	854.78	835.40	-1.428	.08	803.92	792.52	-2.266	.01	638.19	650.59	1.795	**.04***
**0.55**	881.47	851.49	-2.510	.01	826.73	808.19	-3.434	.008	654.88	659.28	0.771	.22
**0.65**	914.11	870.20	-2.782	.006	853.48	823.64	-5.271	.000	669.94	668.80	-0.178	.43
**0.75**	947.98	887.32	-3.791	.000	879.07	837.82	-6.035	.000	694.03	680.32	-1.830	.04
**0.85**	1004.78	902.20	-4.961	.000	919.73	850.95	-7.158	.000	723.73	691.85	-3.608	.001
**0.95**	1095.37	918.30	-7.064	.000	995.98	865.55	-7.953	.000	799.25	702.34	-7.875	.000

Supplementary Table 3. Individual participants' MRTs for audiovisual trials (in ms) and the combined AS+VS CDF computed at each quantile for each age group.

5-6 years old

Participants	1		2		3		4		5		6		7		8		9	
Quantile	MRTs	AS+VS	MRTs	AS+VS	MRTs	AS+VS	MRTs	AS+VS	MRTs	AS+VS	MRTs	AS+VS	MRTs	AS+VS	MRTs	AS+VS	MRTs	AS+VS
0.05	731.50	794.25	698.00	758.80	718.70	688.50	710.25	645.10	697.75	620.15	819.00	745.80	592.00	521.80	827.25	812.00	751.75	740.95
0.15	781.00	809.42	773.75	775.80	730.73	743.65	735.50	732.63	747.25	649.80	836.00	815.00	629.60	600.26	864.75	917.00	766.17	783.40
0.25	788.00	819.48	806.00	796.20	736.00	802.12	743.50	748.50	754.83	685.49	853.00	836.00	652.00	635.68	915.25	961.00	823.00	814.50
0.35	800.00	830.24	843.50	819.39	782.20	810.43	764.50	753.75	769.17	691.19	869.00	876.40	670.00	666.97	952.75	981.20	835.75	835.07
0.45	812.63	847.53	852.83	840.37	830.00	824.26	785.17	768.35	774.25	706.62	879.67	889.33	697.80	672.79	969.25	986.42	873.25	841.53
0.55	832.50	853.88	893.83	862.93	869.20	836.18	786.17	801.50	799.00	738.48	885.33	920.43	728.20	685.68	978.50	991.98	898.75	852.69
0.65	861.00	868.01	926.50	898.80	889.80	840.82	791.00	819.95	836.25	771.22	886.00	940.20	754.25	696.53	994.50	1002.13	920.25	863.24
0.75	885.75	874.15	952.75	929.20	918.00	863.82	840.50	851.13	873.00	793.73	953.00	969.99	797.33	706.18	1014.75	1010.27	982.50	868.35
0.85	944.00	901.24	963.00	961.20	949.00	881.97	922.17	852.84	940.50	807.01	986.00	985.22	858.40	714.63	1108.25	1018.28	1041.25	869.00
0.95	1207.25	921.82	1056.75	982.05	1117.20	899.04	949.33	879.89	1002.75	817.61	1103.00	999.51	868.80	718.55	1271.00	1019.45	1103.50	882.24

Participants	10		11		12		13		14		15		16		17		18	
Quantile	MRTs	AS+VS	MRTs	AS+VS	MRTs	AS+VS	MRTs	AS+VS	MRTs	AS+VS	MRTs	AS+VS	MRTs	AS+VS	MRTs	AS+VS	MRTs	AS+VS
0.05	651.80	636.20	826.50	953.00	634.20	574.50	703.40	674.27	819.25	809.60	740.00	806.25	754.40	722.40	904.70	904.10	520.20	536.00
0.15	728.60	656.20	869.45	964.33	665.00	658.64	721.13	703.07	836.00	845.60	790.25	882.00	768.45	756.40	949.45	928.30	841.50	648.91
0.25	753.00	696.33	881.25	970.80	686.00	680.01	751.00	718.00	852.50	884.05	815.50	886.63	769.75	774.12	1024.00	944.50	869.00	840.30
0.35	809.80	732.60	902.10	977.69	726.40	710.20	798.40	764.67	864.25	885.71	847.17	900.86	789.20	785.66	1041.95	964.20	897.20	947.00
0.45	860.00	765.00	944.25	984.59	748.80	733.88	869.60	768.45	885.17	897.48	891.50	914.79	874.95	801.93	1053.45	993.10	912.80	969.00
0.55	908.80	792.67	1020.30	986.86	772.20	736.87	902.80	772.11	888.67	902.75	919.17	928.51	886.65	836.41	1071.60	1011.80	946.00	969.99
0.65	966.00	832.33	1082.75	998.05	794.60	745.97	979.60	780.57	902.17	933.35	928.25	944.48	917.40	857.72	1097.70	1019.68	988.40	1005.45
0.75	987.00	862.71	1137.50	1007.98	803.00	751.43	1003.00	785.37	905.67	952.78	965.00	963.80	1007.25	894.01	1217.25	1027.11	1002.00	1022.24
0.85	1008.20	890.19	1279.40	1016.91	834.40	757.11	1030.00	791.14	969.25	969.19	998.50	980.87	1065.65	923.71	1295.80	1034.54	1029.20	1056.60
0.95	1127.00	908.47	1440.00	1025.08	835.80	769.37	1177.00	816.17	1132.75	989.69	1036.75	997.03	1159.95	936.72	1446.45	1053.79	1202.50	1069.42

5-6 years old						
**Participants**	**19**		**20**		**21**	
**Quantile**	**MRTs**	**AS+VS**	**MRTs**	**AS+VS**	**MRTs**	**AS+VS**
**0.05**	720.60	547.15	784.70	735.00	718.50	736.31
**0.15**	754.50	786.39	785.73	768.68	734.00	750.11
**0.25**	780.17	817.09	885.00	783. 71	764.00	772.92
**0.35**	795.07	847.22	892.40	813.67	783.17	791.74
**0.45**	809.65	860.30	902.80	861.59	806.00	806.00
**0.55**	837.15	869.05	916.60	900.54	855.50	825.17
**0.65**	852.65	871.27	980.87	948.89	904.25	847.80
**0.75**	902.50	877.91	1014.33	969.95	917.75	876.63
**0.85**	933.45	884.55	1039.80	995.71	950.63	901.16
**0.95**	999.65	893.22	1053.80	1027.29	969.75	915.57

5% = *n* = 6 (28.57%)

15%= *n* =10 (47.61 %)

25%= *n* =10 (47.61%)

35%= *n* =10 (47.61%)

45%= *n* =8 (38 %)

55%= *n* =8 (38%)

7-8 years old																		
**Participants**	**1**		**2**		**3**		**4**		**5**		**6**		**7**		**8**		**9**	
**Quantile**	**MRTs**	**AS+VS**	**MRTs**	**AS+VS**	**MRTs**	**AS+VS**	**MRTs**	**AS+VS**	**MRTs**	**AS+VS**	**MRTs**	**AS+VS**	**MRTs**	**AS+VS**	**MRTs**	**AS+VS**	**MRTs**	**AS+VS**
**0.05**	623.50	672.75	724.80	624.20	704.00	739.53	752.50	763.17	714.50	680.17	724.00	735.30	775.40	676.40	685.25	632.83	774.00	751.40
**0.15**	734.50	720.28	782.00	812.20	721.13	756.31	759.00	779.24	764.00	741.50	774.10	743.05	801.30	717.73	710.75	704.13	792.00	766.20
**0.25**	761.25	747.55	836.00	831.81	733.88	769.06	773.25	804.52	794.25	768.54	794.00	754.61	802.00	746.45	738.67	716.88	803.70	780.58
**0.35**	792.00	753.87	858.80	853.30	762.67	769.96	798.75	827.05	831.00	783.02	813.90	772.81	802.70	763.18	754.83	724.96	813.90	795.24
**0.45**	803.70	771.32	896.20	865.96	769.25	781.46	823.63	835.74	861.25	791.00	819.90	785.89	812.07	783.43	773.00	736.17	835.50	818.15
**0.55**	813.90	789.74	935.40	894.49	778.50	786.56	854.38	849.75	898.00	813.77	837.60	796.99	850.33	802.37	785.75	743.59	852.75	819.90
**0.65**	844.00	806.79	972.20	922.12	799.00	792.86	885.50	865.99	911.25	832.00	852.30	818.12	885.60	816.70	799.33	757.14	874.00	838.76
**0.75**	881.75	823.74	1002.00	934.79	824.67	802.30	923.75	871.72	936.75	845.63	861.50	843.33	969.00	840.67	830.33	767.52	886.75	852.51
**0.85**	891.00	839.46	1056.60	939.34	856.75	808.61	989.75	888.17	978.25	863.70	881.20	876.68	976.40	860.50	877.50	777.89	907.75	860.42
**0.95**	953.25	849.80	1086.80	951.29	868.75	818.43	1000.50	906.57	1066.00	886.65	976.00	896.77	1079.60	878.96	915.00	788.42	944.50	869.34
**Participants**	**10**		**11**		**12**		**13**		**14**		**15**		**16**		**17**		**18**	
**Quantile**	**MRTs**	**AS+VS**	**MRTs**	**AS+VS**	**MRTs**	**AS+VS**	**MRTs**	**AS+VS**	**MRTs**	**AS+VS**	**MRTs**	**AS+VS**	**MRTs**	**AS+VS**	**MRTs**	**AS+VS**	**MRTs**	**AS+VS**
**0.05**	793.40	819.20	567.85	530.70	591.80	605.40	610.25	608.60	723.00	697.00	676.50	702.20	637.60	674.00	677.50	674.60	587.83	608.80
**0.15**	838.80	829.00	697.45	765.60	619.60	634.30	672.50	703.74	747.75	737.77	727.50	712.60	661.60	704.00	765.25	722.00	603.60	619.34
**0.25**	852.67	857.70	852.75	853.25	636.00	653.64	714.50	718.14	772.75	749.86	746.00	736.99	669.00	735.75	852.25	751.62	613.20	625.79
**0.35**	882.40	903.33	902.85	887.90	681.73	663.62	760.50	720.63	823.75	761.95	764.75	785.48	691.60	755.20	864.25	752.83	618.30	633.36
**0.45**	902.80	933.90	924.60	905.18	698.87	678.72	802.50	743.29	852.25	768.38	790.25	809.14	711.00	769.68	869.25	761.73	618.90	641.59
**0.55**	938.60	967.34	968.80	951.82	704.27	693.85	848.75	763.99	861.00	771.72	827.75	829.14	734.33	780.59	888.38	772.87	646.50	649.54
**0.65**	972.80	969.69	1003.15	979.89	720.07	714.35	869.25	779.31	877.50	790.91	857.25	853.37	762.00	792.71	923.13	785.53	666.17	659.56
**0.75**	1020.00	976.83	1099.50	1004.77	735.00	749.26	882.75	785.51	910.00	813.99	878.00	868.79	769.00	802.30	947.88	805.41	679.00	671.21
**0.85**	1169.40	988.24	1220.05	1028.06	809.20	774.48	907.00	790.26	936.25	820.95	886.50	871.42	809.20	809.97	1019.25	827.91	694.00	685.18
**0.95**	1330.00	997.63	1301.60	1051.08	859.20	786.96	1182.25	800.34	1099.75	845.40	998.75	880.99	845.60	833.58	1157.25	840.98	777.25	690.01

7-8 years old														
**Participants**	**19**		**20**		**21**		**22**		**23**		**24**		**25**	
**Quantile**	**MRTs**	**AS+VS**	**MRTs**	**AS+VS**	**MRTs**	**AS+VS**	**MRTs**	**AS+VS**	**MRTs**	**AS+VS**	**MRTs**	**AS+VS**	**MRTs**	**AS+VS**
**0.05**	742.65	822.30	622.25	623.25	723.80	682.40	584.50	678.00	768.25	793.75	791.27	855.13	684.75	677.00
**0.15**	800.40	852.30	641.38	644.00	751.90	755.80	672.25	684.79	769.75	802.74	820.07	867.88	699.17	718.00
**0.25**	843.50	869.36	660.25	653.60	761.00	769.43	718.25	699.22	801.50	816.57	835.00	880.63	707.00	743.50
**0.35**	869.80	881.42	709.75	663.20	774.87	775.93	731.00	705.02	811.00	839.50	855.60	888.41	731.00	752.75
**0.45**	897.25	897.27	719.25	668.71	785.00	785.12	752.25	721.97	827.50	852.47	885.80	899.89	777.50	766.33
**0.55**	930.40	916.29	732.00	678.79	786.10	786.65	764.25	740.12	860.75	866.69	905.00	912.30	815.00	779.63
**0.65**	999.65	937.44	764.25	685.07	833.55	795.59	809.75	751.28	894.50	882.42	918.07	927.97	852.25	786.96
**0.75**	1028.25	962.46	801.75	691.33	881.25	809.96	842.38	755.73	932.00	889.04	919.00	947.34	864.25	796.53
**0.85**	1062.90	983.59	852.75	702.00	1010.95	825.06	859.38	763.20	1028.00	908.73	932.80	954.53	894.75	808.95
**0.95**	1083.75	996.95	903.75	712.67	1157.55	843.58	897.63	770.81	1178.75	929.07	1019.20	979.09	981.75	819.52

5% = *n*= 15 (60%)

15%= *n*= 16 (64%)

25%= *n*= 14 (56%)

35%= *n*= 11 (44%)

45%= *n*= 10 (40%)

55%= *n*= 8 (32%)

9-10 years old																				
**Participants**	**1**		**2**		**3**		**4**		**5**		**6**		**7**		**8**		**9**		**10**	
**Quantile**	**MRTs**	**AS+VS**	**MRTs**	**AS+VS**	**MRTs**	**AS+VS**	**MRTs**	**AS+VS**	**MRTs**	**AS+VS**	**MRTs**	**AS+VS**	**MRTs**	**AS+VS**	**MRTs**	**AS+VS**	**MRTs**	**AS+VS**	**MRTs**	**AS+VS**
**0.05**	591.80	639.00	585.25	585.20	601.50	601.18	621.20	562.00	612.60	605.50	431.40	542.25	571.25	603.70	588.50	634.11	485.25	568.02	586.70	492.75
**0.15**	628.00	660.00	605.80	596.20	602.38	618.47	634.40	642.20	652.20	655.75	562.00	571.17	590.75	619.02	614.75	644.43	597.75	581.87	596.90	618.69
**0.25**	636.00	668.31	635.00	635.00	605.50	640.60	651.33	675.07	663.00	669.00	585.00	603.21	603.42	622.28	646.50	670.19	612.63	589.13	602.30	624.45
**0.35**	656.53	673.12	658.80	651.46	618.17	668.28	684.27	709.45	668.60	672.62	590.53	618.32	611.92	632.51	666.17	696.28	625.00	608.51	602.90	631.48
**0.45**	671.04	681.36	674.12	652.78	623.00	672.04	698.40	761.72	669.80	678.33	605.80	629.16	620.88	635.64	694.50	732.46	639.00	618.50	616.33	635.47
**0.55**	680.56	686.00	683.08	659.71	659.75	683.15	762.20	786.21	685.13	684.05	617.20	641.96	632.13	644.44	727.50	750.64	651.75	624.61	629.00	639.00
**0.65**	686.30	691.43	697.47	667.84	668.83	694.71	822.80	790.38	687.13	690.96	618.60	651.57	640.00	657.28	743.50	759.55	677.25	635.89	635.50	645.86
**0.75**	687.00	701.75	719.00	685.79	682.33	706.85	837.00	809.85	703.00	698.12	635.00	662.83	676.75	669.07	764.75	791.21	726.75	648.05	672.75	651.33
**0.85**	701.40	713.67	742.40	704.18	689.75	718.12	913.00	823.68	786.40	707.18	651.80	674.13	710.50	685.84	827.25	812.79	751.50	659.65	698.25	652.71
**0.95**	863.60	724.27	779.20	714.82	789.50	718.85	1016.60	835.06	886.80	718.82	665.80	685.27	748.75	701.40	952.50	834.36	789.75	670.37	786.00	662.82

**Participants**	**11**		**12**		**13**		**14**		**15**		**16**		**17**		**18**	
**Quantile**	**MRTs**	**AS+VS**	**MRTs**	**AS+VS**	**MRTs**	**AS+VS**	**MRTs**	**AS+VS**	**MRTs**	**AS+VS**	**MRTs**	**AS+VS**	**MRTs**	**AS+VS**	**MRTs**	**AS+VS**
**0.05**	521.50	556.25	534.20	568.13	588.25	593.25	567.50	576.75	570.27	602.25	589.00	600.07	505.25	518.20	606.25	606.25
**0.15**	535.75	577.00	545.20	569.07	601.50	610.25	585.00	604.14	585.07	614.25	613.75	615.37	530.75	538.80	631.00	627.00
**0.25**	572.25	585.50	553.00	570.00	602.50	618.76	601.50	615.72	586.00	619.25	639.50	634.95	549.17	558.80	643.50	635.16
**0.35**	590.10	601.08	568.27	584.00	611.00	625.45	602.75	624.37	602.40	631.25	703.25	653.75	552.83	567.32	652.10	637.25
**0.45**	600.30	614.15	571.40	598.87	623.00	633.19	616.33	632.09	615.00	651.25	735.25	667.25	560.50	569.25	652.70	647.08
**0.55**	610.00	618.78	582.60	607.63	631.00	634.84	619.63	634.66	622.40	625.75	760.75	693.30	568.10	569.71	665.00	655.64
**0.65**	618.25	635.14	585.73	618.16	640.10	635.79	644.00	639.12	645.60	663.00	790.13	726.10	568.70	572.70	689.00	662.93
**0.75**	635.50	645.00	618.00	619.68	650.30	642.38	705.50	645.77	685.00	671.34	814.88	744.94	577.50	580.00	701.50	671.69
**0.85**	710.00	660.91	619.40	629.13	668.25	650.63	723.25	651.19	701.40	685.22	819.63	751.75	602.50	584.59	710.50	687.86
**0.95**	806.25	672.46	685.60	638.11	707.25	654.04	748.75	653.70	729.20	697.38	894.25	773.17	706.75	585.95	794.00	701.18

5% = *n*= 12 (66.66%) 15%= *n*= 14 (77.77%) 25%= *n*= 14 (77.77%) 35%= *n*= 13 (72.22%) 45%= *n*= 14 (77%) 55%= *n*= 13 (72.22%)
